# *Ilex kaushue* and Its Bioactive Component 3,5-Dicaffeoylquinic Acid Protected Mice from Lipopolysaccharide-Induced Acute Lung Injury

**DOI:** 10.1038/srep34243

**Published:** 2016-09-29

**Authors:** Yu-Li Chen, Tsong-Long Hwang, Huang-Ping Yu, Jia-You Fang, Kowit Yu Chong, Yao-Wen Chang, Chun-Yu Chen, Hsuan-Wu Yang, Wen-Yi Chang, Pei-Wen Hsieh

**Affiliations:** 1 Graduate Institute of Biomedical Sciences, College of Medicine, Chang Gung University, Taoyuan, Taiwan; 2 Graduate Institute of Natural Products, College of Medicine, Chang Gung University, Taoyuan, Taiwan; 3 Chinese Herbal Medicine Research Team, Healthy Aging Research Center, Chang Gung University, Taoyuan, Taiwan; 4 Research Center for Industry of Human Ecology and Research Center for Chinese Herbal Medicine, Chang Gung University of Science and Technology, Taoyuan, Taiwan; 5 Department of Anesthesiology, Chang Gung Memorial Hospital, Taoyuan, Taiwan; 6 School Medicine, College of Medicine, Chang Gung University, Taoyuan, Taiwan; 7 Department of Medical Biotechnology and Laboratory Science, College of Medicine, Chang Gung University, Taoyuan, Taiwan; 8 Department of Thoracic Medicine, Chang Gung Memorial Hospital at Linkou, Taoyuan, Taiwan

## Abstract

Acute lung injury (ALI) is a severe respiratory disease with high mortality rates worldwide. Recent reports suggest that human neutrophil elastase (HNE) plays a key role in the inflammatory response that is characteristic of ALI, which indicates that the development of HNE inhibitors could be an efficient treatment strategy. In the current study, an enzyme-based screening assay was used to identify effective HNE inhibitors from a number of traditional Chinese medicines (TCMs). Among them, a water extract of *Ilex kaushue* (IKWE) effectively inhibited HNE activity (IC_50_, 11.37 ± 1.59 μg/mL). Using bioactivity-guided fractionation, one new compound and 23 known compounds were identified. Compound **6** (identified as 3,5-dicaffeoylquinic acid; 3,5-DCQA) exerted the most potent and selective inhibitory effect on HNE activity (IC_50_, 1.86 ± 0.06 μM). In a cell-based assay, 3,5-DCQA not only directly reduced superoxide generation and elastase activity but also attenuated the Src family kinase (SRKs)/Vav signaling pathway in N-formyl-L-Met-L-Leu-L-Phe (fMLF)-stimulated human neutrophils. In an animal disease model, both 3,5-DCQA and standardized IKWE protected against lipopolysaccharide-induced ALI in mice, which provides support for their potential as candidates in the development of new therapeutic agents for neutrophilic inflammatory diseases.

ALI and its more severe form, acute respiratory distress syndrome (ARDS), are inflammatory diseases triggered by direct and indirect pathogenic factors, such as sepsis, pneumonia, inhalation injury and trauma^1^. Lipopolysaccharide (LPS) is an endotoxin that plays a pathological determinant role in sepsis-related ALI^2^. In the majority of cases, ALI and ARDS result in respiratory failure leading to high mortality. No effective therapeutic agents are available for ALI, and technical ventilation and supportive care constitute the primary approaches to avoid underlying complications^1^,^3^,^4^, highlighting the urgent requirement for novel treatment strategies and medicines.

Neutrophils form the first line of defense against pathogens in innate immunity mainly through phagocytosis. Further invasion of pathogens is prevented with the release of reactive oxygen species (ROS), serine proteases, and neutrophil extracellular traps^5^. In ALI pathology, circulating neutrophils are recruited and activated by chemokines and cytokines from alveolar macrophages and alveolar-type II epithelial cells, triggering neutrophil serine protease release, alveolar edema and impaired oxygenation. Neutrophil elastase (NE) is one of the serine proteases released from activated neutrophils that cause pulmonary damage through hydrolysis of elastin-rich connective tissue. Additionally, NE acts as an inflammatory mediator and contributes to the migration and activation of neutrophils through effects on alveolar macrophage and lung epithelial cells^6^. α_1_-Antitrypsin, an endogenous secretory elastase inhibitor abundant in the peripheral alveolar region, naturally protects lung tissue from proteolysis by elastase. Enhanced NE activity has been observed due to inactivation of α1-antitrypsin under conditions of increased oxidative stress resulted from neutrophils^4^,^7^. The development of NE inhibitors is therefore considered an effective therapeutic strategy for ALI^3^,^4^.

Botanical products and TCM are recognized as important sources of novel drugs^8^,^9^. In an attempt to identify NE inhibitors, 22 TCM extracts were prepared and their inhibitory effects on human neutrophil elastase (HNE) activity evaluated. Among these, the *I. kaushue* water extract (IKWE) inhibited HNE activity with an IC_50_ value of 11.37 ± 1.59 μg/mL. *I. kaushue*, syn *I. kudingcha* (also known as Kudingcha) is an evergreen tree found in China^10^. The leaves have been used as a daily beverage and herbal medicine in TCM for nearly two thousand years^11^. Triterpenoids, polyphenols, cyanoglucosides and essential oils are the major metabolites of *I. kaushue*^12^,^13^. Traditionally, the plant has beneficial physiological effects, including thirst quenching, elimination of phlegm for resuscitation, and removal of mucus from the lung^14^. Recent studies have additionally revealed anticancer, antidiabetes, antiobesity and antioxidant bioactivities^12^,^13^,^15^,^16^,^17^. In the current investigation, we focused on evaluating the beneficial effects of *I. kaushue* and its bioactive component on acute lung injury (ALI), both *in vitro* and *in vivo*.

## Results

### Chemical components isolated from *I. kaushue* water extracts (IKWE) via bioactivity-guided fractionation

**Figure 1:**
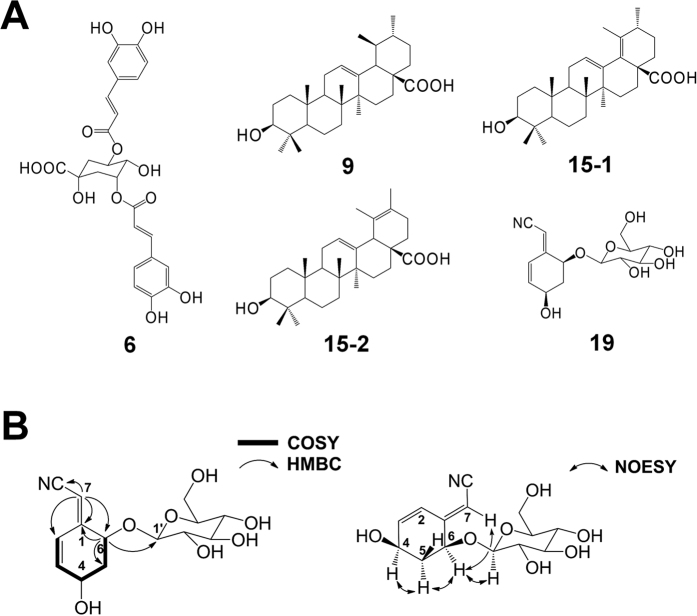
Structure of selected isolates and synthetics. (**A**) 3,5-DCQA (**6**), ursolic acid (**9**), randialic acid B (**15**-**1**), sanguisorbigenin (**15**-**2**), and menisdaurin F (**19**); (**B**) Selected COSY, HMBC and NOE correlation of menisdaurin F.

Using bioassay-directed fractionation, ninteen compounds, including polyphenols (**1**-**8**), triterpenoid saponins (**9**-**15**), cyanoglucosides (**17**-**19**) and phytosteroid (**16**) (Fig. 1A and Supplementary information Fig. S1) were isolated from IKWE. Among these, compound **19** was new, while compounds **1**~**8** were identified as caffeoylquinic acids (CQA) or dicaffeoylquinic acids (DCQA) based on the position and number of caffeoyl groups conjugated with quinic acid. Saccharides of **11**, **14**, and **15** were further hydrolyzed to generate aglycons (**11**-**1**, **11**-**2**, **14**-**1**, **15**-**1**, and **15**-**2**) for proposing structure and activity relationships (SAR). All known compounds were identified by comparing their physical and spectral data with the values provided in the literatures.

### Structural elucidation of menisdaurin F (compound 19)

Compound **19**, [α]^23^_D_: −63.6° (*c* = 0.05, MeOH), was obtained as a colorless powder. The molecular formula, C_14_H_19_NO_7_, assigned based on HRESIMS data (336.1048 *m*/*z*, [M + Na]^+^), implied six unsaturation degrees. The presence of a hydroxyl group was suggested based on the absorption band at 3392 cm^−1^ in the IR spectrum. The characteristic bands at 2220 and 1621 cm^−1^ in the IR spectrum, ^13^C NMR signals at *δ*_C_ 93.7 (d), 117.9 (s), 124.5 (d), 143.3 (d), 159.1 (s) and ^1^H NMR signals at *δ*_H_ 5.77 (s), 6.24 (d, 10.0), 6.57 (dd, 10.0, 2.0), together with ultraviolet absorption at *λ*_max_ 256 nm, signifying the presence of α, β, γ, δ-unsaturated nitrile^18^,^19^,^20^. NMR signals at *δ*_H_ 3.66 (dd, 11.6, 8.0), 3.86 (d, 11.6), 4.48 (d, 8.0); *δ*_C_ 62.6 (t) and 102.6 (d) suggested the presence of a glucopyranosyl group. Comparison of 1D and 2D NMR spectra revealed that compound **19** is a structural isomer of menisdaurin (**18**) (Fig. 1B, Supplementary information Figs S2–S8 and Table S1)^19^,^20^,^21^. The strong NOE (nuclear overhauser effect) correlations between H-4 and H-5a, H-5a and H-6, and H-6 and H-1’ demonstrate that compound **19** shares the same orientation of H-4 and H-6 with menisdaurin. Additionally, NOE correlations between H-6 and H-7 implied an *E*-configuration of the C-1/C-7 double bond in compound **19**^19^,^22^. The downfield shift of the H-2 signal (calculated as 0.25 ppm) additionally supported the presence of the *E*-configuration^19^. Based on the collective data, the structure of **19** was determined as an *E*-form isomer of menisdaurin (**18**), designated menisdaurin F.

### 3,5-DCQA (compound 6) showed potent and selective inhibition of HNE activity

To identify the bioactive components and their specificities, enzyme inhibition assays involving human and bovine serine proteases were performed (Table 1). Among the isolates, 3,5-DCQA (**6**) exerted the most potent and selective inhibitory effect on HNE activity with an IC_50_ value of 1.86 ± 0.06 μM. All triterpenoid saponins (**10**-**15**) were non-active, while aglycons (**11**-**2** and **15**-**2**) and ursolic acid (**9**) exerted non-selective inhibitory effects on serine proteases.

### 3,5-DCQA reduced superoxide anion (O_2_^.−^) production and NE activity in fMLF-activated human neutrophils

O_2_^.−^ and NE from activated neutrophils cause alveolar damage in response to acute inflammatory conditions in ALI. Therefore, the effects of all isolates and semi-synthetics on O_2_^.−^ generation and NE release were determined using fMLF as an inducer in human neutrophils (Table 2). Our results showed that 3,5-DCQA inhibited O_2_^.−^ generation and NE activity with IC_50_ values of 1.92 ± 0.54 and 12.02 ± 0.60 μM, respectively (Supplementary information Table S2). Three triterpenoids, **9**, **15**-**1** and **15**-**2**, exhibited good inhibitory effects on O_2_^.−^ generation and NE release as well. However, triterpenoid saponins (compounds **10**-**15**) showed no or weak inhibitory effects.

### 3,5-DCQA attenuated the activation of SFKs and Vav in fMLF-induced neutrophils

**Figure 2:**
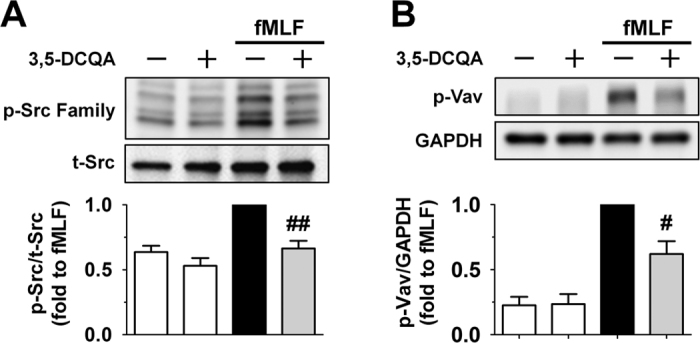
3,5-DCQA inhibited the phosphorylation of Src family kinase (Src) and Vav in fMLF-activated human neutrohpils. Human neutrophils were pre-incubated with dimethylsulfoxide (DMSO) or 3,5-DCQA (10 μM) for 5 min before stimulation with or without fMLF (0.1 μM) for another 0.5 min. All the Western blotting experiments were performed under the same condition. After transferring the blots onto nitrocellulose membranes, the targeted blots were cropped immediately according to referenced indicating markers. The targeted proteins were immunoblotted with its specific monoclonal antibody. (**A**) Src family kinases; (**B**) Vav. Targeted bands were analyzed using a densitometer and normalized to the corresponding total protein or glyceraldehyde 3-phosphate dehydrogenase (GAPDH). The densitometric data were presented as mean ± S.E.M. (n = 3–4). Compared with fMLF group: ^**#**^*p* < 0.05 and ^**##**^*p* < 0.01.

To investigated whether 3,5-DCQA modulated neutrophil activity through intracellular signaling pathway, the activation of SFKs, Vav, Akt and MAPKs were evaluated by Western blot. The results indicated fMLF triggered the phosphorylation of signaling proteins, and 3,5-DCQA was able to reduce phosphorylation of SFKs and Vav, but not Akt and MAPKs (Fig. 2 and Supplementary information Fig. S9).

### Establishment of CMC (chemistry, manufacturing and controls)

**Figure 3:**
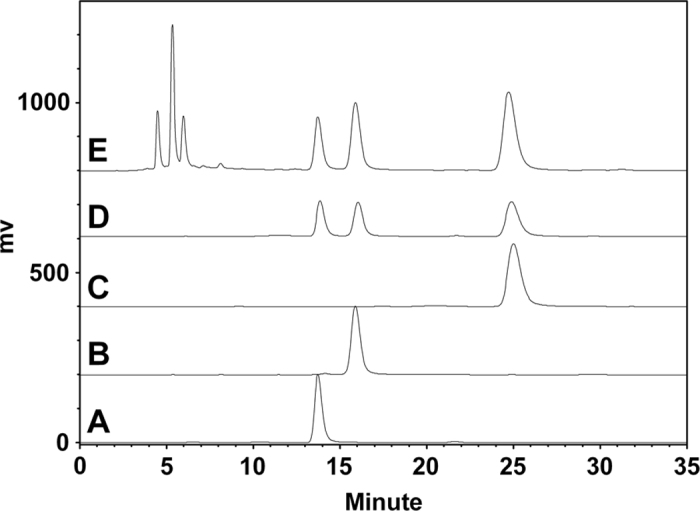
HPLC fingerprint chromatography and standard references of IKWE. (**A**) 3,4-DCQA, 100 μg/mL, rt 13.5 min; (**B**) 3,5-DCQA, 100 μg/mL, rt 15.9 min; (**C**) 4,5-DCQA, 200 μg/mL, rt 25.0 min; (**D**) Combination of 3,4-DCQA, 3,5-DCQA, and 4,5-DCQA at 50, 50, 100 μg/mL respectively; (**E**) IKWE, 2 mg/mL. Injection volume, 20 μL; Detection wavelength, 326 nm; Flow rate, 0.8 mL/min; Mobile phase, 42.5% MeOH solution with 1% formic acid.

CMC data are essential to maintain the quality of botanical products in manufacturing. Accordingly, SOPs (standard operating procedures) and quality control were performed for the IKWE preparation. In HPLC fingerprints, three distinct peaks were identified as 3,4-DCQA (**4**), 3,5-DCQA (**6**) and 4,5-DCQA (**7**), compared to the respective pure compounds (Fig. 3). We defined and quantified these DCQAs as chemical reference standards based on calibration curves (Supplementary information Fig. S10). Biological identification was additionally validated with the HNE activity assay. The yield for three batches of IKWE was generally over 35% (Supplementary information Fig. S11), and amounts of **4**, **6**, and **7** obtained were 3.76 ± 0.26%, 4.70 ± 0.13% and 8.85 ± 0.15%, respectively. These data indicated that 3,5-DCQA served as the main bioactive component in KSWE. Besides, batches of IKWE exerted an inhibitory effect on HNE activity with an IC_50_ value of 10.50 ± 0.48 μg/mL, signifying the stable quality of bioactivity and chemical composition.

### Toxicity Evaluation of IKWE

**Figure 4:**
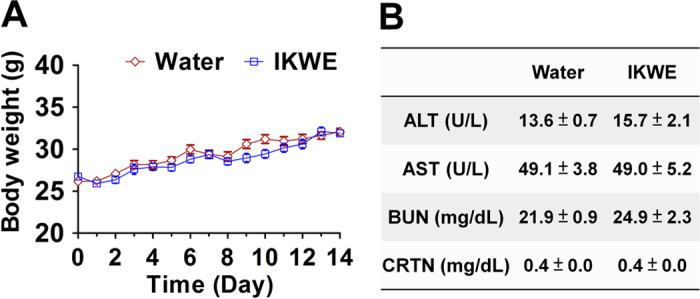
Acute oral toxicity evaluation of IKWE. IKWE were dissolved with ddH_2_O to give IKWE solution. Body weights of mice received vehicle or IKWE solution through gavage were observed for 14 days. Serum was obtained 24 hours after gavage for chemical parameter determination. (**A**) Curves of body weight growth in vehicle-received or IKWE-received mice (n = 10 for each group); (**B**) Examination of liver and kidney functions between vehicle and IKWE groups. ALT, alanine transaminase; AST, aspartate aminotransferase; CRTN, creatinine; BUN, blood urea nitrogen. Data were presented as mean ± S.E.M. (n = 10).

Safety of botanical drugs is another important concern. To evaluate the toxicity of IKWE, acute oral toxicity studies were performed at a high dose of 10.0 g/kg^23^. All mice receiving IKWE with LD_50_ values higher than 10.0 g/kg survived. Moreover, no significant differences were observed with regard to movement and body weight growth in mice, suggesting no adverse effects (Fig. 4A). Organ toxicity was further assessed via determination of liver and kidney function (Fig. 4B). The data collectively indicated high safety of IKWE.

### IKWE and 3,5-DCQA protected against LPS-induced ALI in mice

**Figure 5:**
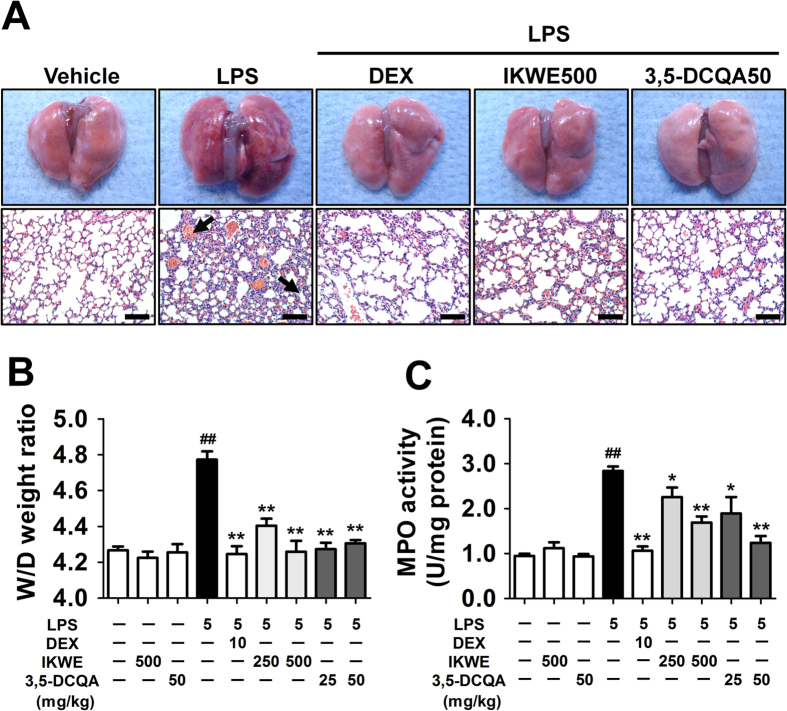
IKWE and 3,5-DCQA attenuated LPS-induced ALI in mice. Mice pretreated with vehicle or drugs intraperitoneally received intratracheal instillation of LPS. After 6 hours, mice were anesthetized and their chests were opened. Whole lungs were obtained immediately and observed for morphological changes. Left lobe was then dissected for histology or MPO activity. Right lobes were applied for W/D weight ratio. (**A**) Morphological observation and histological examination (arrows indicated hemorrhage and infiltrated neutrophils), scale bar = 50 μm; (**B**). (**C**) Lung W/D weight ratio and MPO activity. Data were presented as mean ± S.E.M. (n = 6). Compared with vehicle group: ^**##**^*p* < 0.01; Compared with LPS group: *p < 0.05 and **p < 0.01.

**Figure 6:**
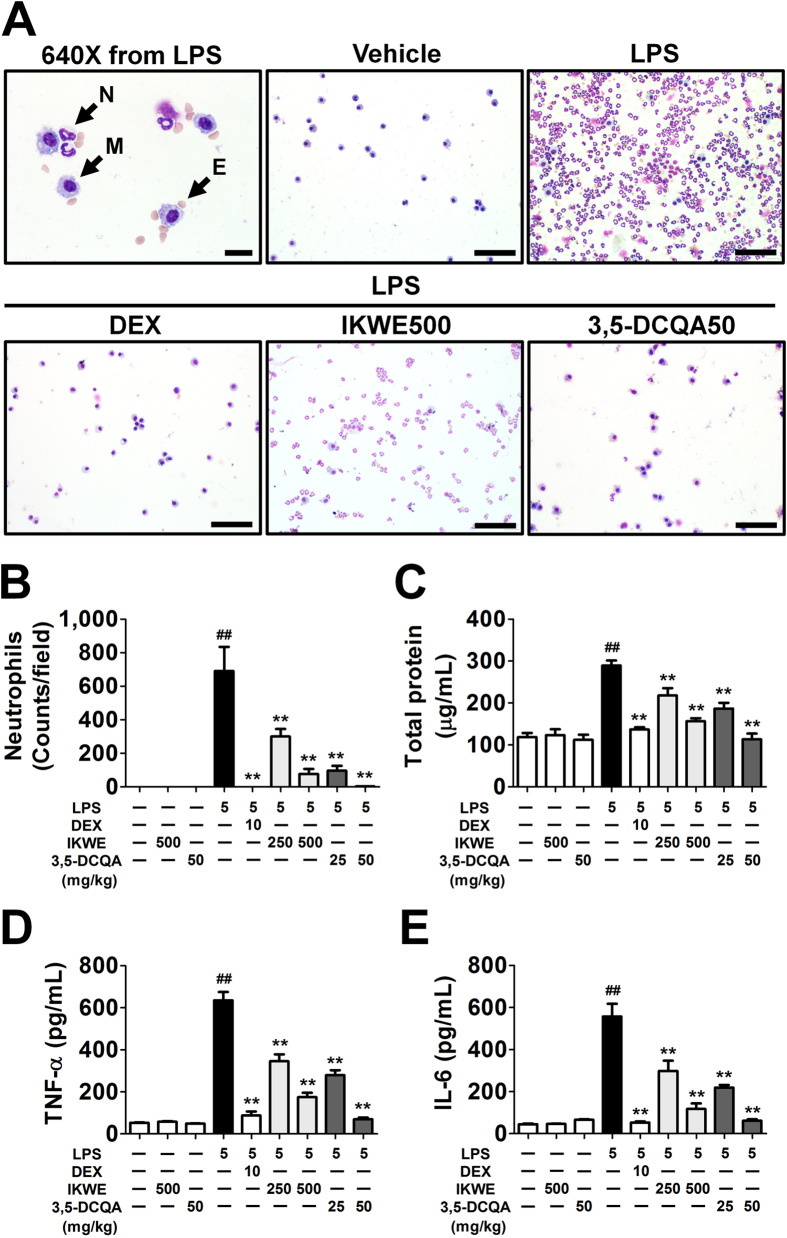
Content analysis of BALF. Mice were anesthetized and inserted with a plastic cannula into the trachea. After clamping the hilum of left lobe, PBS was injected and recovered to give BALF. Supernatant from centrifuged BALF were applied for total proteins, TNF-α and IL-6 measurement. Cell pellets were resuspended and cytospun for population analysis. (**A**) Examination of cell populations, N: neutrophils, M: macrophages, E: erythrocytes (scale bar = 50 μm in ×200 magnification or 10 μm in ×640 magnification); (**B**) Counts of BALF neutrohpils; (**C**) Levels of total proteins; **(D**) Levels of TNF-α; (**E**) Levels of IL-6. Data were presented as mean ± S.E.M. (n = 6). Compared with vehicle group: ^**##**^*p* < 0.01; compared with LPS group: **p < 0.01.

In view of the finding that IKWE and its bioactive component, 3,5-DCQA, exert anti-inflammatory effects against activated neutrophils, both were prepared and their protective effects evaluated in an LPS-induced ALI disease model. Infiltrating neutrophils, thickening of the alveolar wall, lung edema and alveolar hemorrhage are pathological features of ALI^1^,^2^,^3^,^4^. Dark red color and morphological swelling of the lung in LPS-induced ALI mice suggested alveolar hemorrhage and edema (Fig. 5A). In addition to hemorrhage, histological examination revealed infiltrating neutrophils and thickening of the alveolar wall in the LPS-treated group. These pathological features were significantly improved following pretreatment with IKWE (500 mg/kg) or 3,5-DCQA (50 mg/kg). Wet/dry (W/D) weight ratio, myeloperoxidase (MPO) activity and branchalveloar lavage fluid (BALF) were further assessed to confirm the protective effects and determine underlying molecular mechanisms. Our results showed that IKWE and 3,5-DCQA not only improve lung edema but also suppress accumulation of neutrophils in lung tissue (Figs 5B,C and 6A–C). Reduced levels of proinflammatory cytokines (TNF-α and IL-6) were additionally observed (Fig. 6D,E), clearly supporting the protective effects of both IKWE and 3,5-DCQA against LPS-induced ALI in mice. Besides, the post-treatment with 3,5-DCQA was performed to further evaluate its effects against LPS-induced ALI in mice^24^. The results showed that pathological features of ALI were also significantly improved following treatment with 3,5-DCQA (Supplementary information Fig. S12).

## Discussion

ALI is a life-threatening disease for which no effective treatments are available. Botanical drugs are considered to be an important resource for drug development, and they require stringent testing for efficacy, safety and quality^9^. Because of the ability of IKWE to inhibit HNE activity, the protective effects of *I. kaushue* against ALI were investigated, both *in vitro* and *in vivo*. In the *in vivo* studies, dexamethasone was used as a positive control. Although the applied dosage and possible side effects of dexamethasone in clinical settings are controversial, it is still a common positive control in ALI mouse models^25^,^26^,^27^.

Although serine proteases are responsible for several physiological functions in humans, abnormal and excessive levels can cause or promote disease^28^,^29^. Recent experimental and clinical studies have shown that enhanced HNE activity is associated with the degradation of elastin-rich proteins in the pathological progression of ALI. Consequently, HNE is considered to be a promising therapeutic target for treating ALI^4^,^28^,^30^. In our experiments, 3,5-DCQA exhibited high selectivity for HNE among the five serine proteases that were examined. Further exploration of the SAR of the caffeic acid analogues showed that the inhibitory effects on HNE activity of the compounds did not depend on the caffeic acid moiety, but were correlated with the regioselectivity of caffeic acids conjugated to quinic acid (Supplementary information Table S3).

In addition to serine proteases, enhanced oxidative stress also contributes to the pathogenesis of inflammatory diseases^1^,^4^,^30^. The production of superoxide anions from human neutrophils can be reduced through intracellular mechanisms or ROS scavenging agents^4^,^30^. 3,5-DCQA was previously reported to be a ROS-scavenger and to exert anti-oxidant effects^31^. The *ortho*-dihydroxyphenyl moiety significantly promotes ROS scavenging activity via a highly favorable electron acceptance and resonance system^32^,^33^. Our results showed that all caffeoylquinic acid derivatives, together with CA, RA and THBA, potently inhibit O_2_^.−^ generation in neutrophils (Table 2). In contrast, compounds *p*-CA, 3-HCA and FA were non-active because the ortho-dihydroxyphenyl group was absent. Interestingly, the HNE inhibitory effects of 3,5-DCQA decreased in fMLF-induced neutrophils, which was demonstrated by comparing the results of an enzyme inhibition assay. To eliminate the influence of O_2_^.−^ generation, leukotriene B4 (LTB4) was used as an inducer in human neutrophils (Supplementary information Table S2), which led to the recovery of 3,5-DCQA-induced HNE inhibition^34^. These data confirmed that 3,5-DCQA at a high dose (100 μM) was able to inhibit elastase release, myeloperoxidase activity and superoxide production in human neutrophils^35^.

SFKs belong to the family of tyrosine kinases and play important roles in neutrophil activation^36^,^37^. Previous reports have shown that SFKs are responsible for O_2_^.−^ generation and cell migration in fMLF-stimulated neutrophils^36^. In addition, SFKs have been shown to cause elevated expression of TNF-α and chemokines in LPS-induced neutrophils^37^. These effects have been inhibited effectively by PP2, a highly selective SFK inhibitor^36^,^37^. Vav, a member of the guanine nucleotide exchange factors (GEFs) family, causes activation of NADPH oxidase by activating Rac^38^. A previous study revealed that SFKs were able to enhance Vav phosphorylation and activity^39^. Our immunoblotting assay showed that 3,5-DCQA inhibited SFKs and Vav phosphorylation in fMLF-induced neutrophils, which suggests that 3,5-DCQA might attenuate O_2_^.−^ generation, cell migration and TNF-α and chemokine expression in stimulated neutrophils through the SFKs/Vav signaling pathway. Other signaling proteins such as ERK, p38 and Akt are also important in neutrophil activation^36^. However, 3,5-DCQA does not reduce the levels of phosphorylated ERK, p38, and Akt. Based on enzyme-based and cell-based data, we proposed that 3,5-DCQA modulates neutrophil function through intercellular and intracellular mechanisms simultaneously.

Ursolic acid (**9**) was previously reported to inhibit O_2_^.−^ production and NE release from neutrophils through an intracellular mechanism^40^. The findings of the effects of ursolic acid on neutrophils were supported by our enzyme-based and cell-based data. In addition, randialic acid B (**15**-**1**) and sanguisorbigenin (**15**-**2**) exhibited 5-fold greater efficacy than ursolic acid. SARs analysis of triterpenoid saponins (**10**-**15**) and aglycons indicated that their efficacy in fMLF-induced neutrophils was affected by sugar moiety and structural modifications at the E-ring. For example, triterpenoid saponins showed weak or no inhibitory effects on O_2_^.−^ generation and NE release, indicating that saccharides in saponins are unfavorable for bioactivity in human neutrophils. Further, when the carboxylic acid at C-15 was cyclized with a hydroxyl group at C-20 to form a δ-lactone ring (**11**-**1** and **11**-**2**), bioactivity vanished, suggesting that carboxylic acid is necessary for this activity. Conversely, the presence of a double bond at C-18/C-19 or C-19/C-20 (**15**-**1** and **15**-**2**) enhanced human neutrophil activity.

Increased oxidative stress has also been reported to enhance elastase activity by inactivating α_1_-antitrypsin^4^,^7^. Therefore, a combination of HNE inhibitors and free radical scavengers is considered to be an effective strategy for treating ALI. A previous study reported that better therapeutic effects could be achieved with a combined treatment of sivelestat sodium and edaravone in LPS-induced ALI in rats^41^. In our current experiments, enhanced protective effects were observed following treatment with IKWE (500 mg/kg) compared to treatment with 3,5-DCQA (25 mg/kg) alone. The amount of 3,5-DCQA in IKWE at a 500 mg/kg dose is ~25 mg/kg. Because caffeoylquinic acid derivatives may improve α_1_-antitrypsin activity via their ROS scavenging ability, our results suggest that there are synergistic ROS-scavenging effects and a protease-antiprotease balance of other caffeoylquinic acid derivatives in the extract.

Neutrophil recruitment into inflammatory tissue by chemokines is a key event in ALI. Neutrophil locomotion includes adhesion and migration through endothelial cells. L-selectin, β_2_-integrin and platelet-endothelial cell adhesion molecule-1 (PECAM-1) are important adhesion proteins and are quickly expressed in activated neutrophils. These adhesion proteins promote neutrophil attachment to endothelial cells and firm their migration^30^. Chlorogenic acid (**3**) is another major chemical component in *I. Kaushue*^12^, and it was able to attenuate the adhesive ability of neutrophils by inhibiting L-selectin cleavage and reducing β_2_ integrin levels, as well as suppressing the expression of PECAM-1 that is induced by LPS in neutrophils^42^. In the same study, chlorogenic acid also exhibited inhibitory effects on fMLF-induced neutrophil migration. In our experiments, both a BALF analysis and histological data showed that neutrophil infiltration in ALI mice was significantly reduced following treatment with IKWE or 3,5-DCQA. However, no difference was found between the IKWE (500 mg/kg) and 3,5-DCQA (25 mg/kg) groups. These findings suggest that the effects of chlorogenic acid on reducing neutrophil recruitment were not apparent in our *in vivo* experiments. Because the effects of 3,5-DCQA on neutrophil recruitment, adhesion and migration are unclear, further evaluation of the effects of 3,5-DCQA is necessary.

Macrophages are also important in inflammatory diseases. In ALI, there is an increased release of inflammatory cytokines from stimulated macrophages^43^. Neutrophil elastase has been shown to stimulate TNF-α and IL-6 expression in macrophages^44^. A previous study demonstrated that 3,5-DCQA was able to reduce TNF-α and IL-6 expression in LPS-stimulated RAW 264.7 cells^45^. In the current study, 3,5-DCQA inhibited neutrophil elastase and reduced BALF inflammatory cytokines *in vitro* and *in vivo*. These suggested that 3,5-DCQA might be able to modulate macrophage function by inhibiting elastase and intracellular mechanisms simultaneously^44^.

Pharmacokinetic properties are important in drug development. Previous studies have reported that although caffeoylquinic acids derivatives have similar chemical properties, their pharmacokinetic parameters are different^46^,^47^,^48^,^49^. Specifically, the terminal elimination half-life (*T*_*1/2z*_) and the mean residence time (*MRT*) of 3,5-DCQA were 227~292 min and 227~438 min, respectively, after oral administration, and 3,5-DCQA was detectable in the plasma for 4 hours after intravenous administration^46^,^47^,^49^. In addition, chlorogenic acid was distributed in lung tissue more than 1.5 hours after oral administration^48^. These studies may be able to illustrate the efficacy of 3,5-DCQA and its possible metabolites *in vivo*; however, further investigation of the bio-distribution of 3,5-DCQA and its possible metabolites in lung tissue is necessary.

CMC data are critical to determine and maintain the quality of botanical products. Original identification, HPLC fingerprints, chemical and biological references and standardized manufacturing procedures are required for CMC. The internal transcribed spacer (ITS) sequence is the determining factor in genomic identification. However, no ITS information is available for *I. kaushue*, excepting a review article published in 2010^50^. To identify the origin of *I. kaushue* from commercial, two gene sequences, *trnS*-*trnG* and *trnH*-*psbA*, were analyzed and compared using the genomic identification database. The results confirmed 100% and 99% sequence identity to *I. kaushue*, respectively. Subsequently, three batches of IKWE (IKWE-1–3) and water extracts from other two commercial Kudingcha materials (KUD-1 and NKUD-1) were prepared using a standardized manufacturing protocol, followed by determination of HPLC fingerprints, extraction yield, amounts of chemical references and biological activity of each extract. The results indicate good quality of each extract with high reproducibility and consistency (Supplementary information Fig. S11).

In addition to anti-oxidative capacity, 3,5-DCQAs and other DCQAs display diverse bioactivities, including hepatoprotection, anti-hyperlipidemia and anti-thrombosis^51^,^52^,^53^. Anti-viral activities against HIV, RSV, H1N1 and HBV have additionally been demonstrated^54^,^55^,^56^,^57^. Here, we identified three abundant DCQAs in IKWE, supporting the possibility of different biological applications. For example, Shuang-Huang-Lian, a TCM preparation composed of *Flos lonicerae* and other two herbs, is applied to treat various acute respiratory infections, such as influenza virus A-induced pneumonia in China^46^,^47^. Chlorogenic acid is the main bioactive component in *F. lonicerae*. A pharmacokinetic study reported that the amount of chlorogenic acid is 50-fold higher than that of 3,4-DCQA and 200-fold higher than that of 3,5-DCQA in rat blood plasma^45^. The CQA amount is comparable to that of DCQA in *I. kaushue*^12^. Considering the anti-inflammatory and broad anti-viral effects of DCQAs, further evaluation of the effects of IKWE on influenza virus A-induced pneumonia is warranted.

In conclusion, we have developed a standard protocol to prepare a water extract of *I. kaushue* with good reproducibility, consistency, and safety. 3,5-DCQA, the bioactive component in *I. kaushue*, modulated neutrophil function not only by directly targeting HNE and ROS but also by inhibiting the SRKs/Vav signaling pathway. Both IKWE and 3,5-DCQA exhibited protective effects against LPS-induced ALI in mice. These data provide support for both IKWE and 3,5-DCQA as candidates in the development of new lead agents to treat ALI.

## Methods

### Genomic identification of *I. kaushue*

Three raw materials of Kudingcha were purchased from two retailers. Two (IK and KUD) were from Huang-De-An (New Taipei, Taiwan) on 2013/11/20. The third (NKUD) was from Da-Ye (Nantou, Taiwan) on 2015/06/01. Samples from IK were prepared for genomic analysis by Bioproduction Engineering Technology Department, Biomedical Technology and Device Research Laboratories, ITR (Hsinchu, Taiwan). Briefly, genomic DNA was obtained and two genes, *trnS*-*trnG* and *trnH*-*psbA*, subsequently sequenced. Sequence identities to *I. Kaushue* were determined as 100% and 99%, respectively, according to the NCBI genome database.

### Preparation and quality control of water extracts

Each raw material sample (20 g) was refluxed twice with 200 mL ddH_2_O for 2 h. The solutions were filtered and concentrated under vacuum to generate crude extracts (IKWE-1~3, KUD-1, and NKUD-1). The specifications of quality control included extraction yield, HPLC fingerprint chromatography, as well as determination of chemical and biological references. To establish HPLC chromatographic fingerprints, each extract (2.0 mg/mL) was dissolved in mobile phase solution (1% formic acid in 42.5% MeOH aqueous solution), filtered through a 0.45 μm membrane filter, and passed through an HPLC system. The Develosil^™^ C30-UG-5 column (4.6 × 250 mm, 5 μm) (Nomura, Japan) was eluted with a mobile phase consisting of 1% formic acid in 42.5% MeOH aqueous solution at a flow rate of 0.8 mL/min. The detector wavelength and injection volume were set at 326 nm and 20 μL, respectively. Chemical reference levels were determined from calibration curves generated at a concentration range of 40 to 120 μg/mL for 3,4-DCQA and 3,5-DCQA or 80 to 240 μg/mL for 4,5-DCQA. The HNE activity assay was further performed for biological validation.

### Bioactivity-guided fractionation

The isolation procedures and 1D/2D NMR and physical properties of isolates and semi-synthetics are described in Supplementary information S1.

**Menisdaurin F** (Supplementary information Figs S2–S8).

Colorless plate; [α]^23^_D_: −63.6° (*c* = 0.05, MeOH); UV λ_max_ (MeOH) nm (log ε): 256 (4.37); IR *v*_max_ (KBr) cm^−1^: 3392, 2924, 2220, 1621, 1018; Mp: 176–177°; ESIMS: *m*/*z* 336.2 [M + Na]^+^; HRESIMS: *m*/*z* 336.1048 [M + Na]^+^(calcd. for C_14_H_9_NO_7_Na, 336.1054); ^1^H NMR (CD_3_OD, 400 MHz) *δ*: 6.57 (1H, dd, *J* = 10.0, 2.0 Hz, H-2), 6.24 (1H, d, *J* = 10.0 Hz, H-3), 5.77 (1H, s, H-7), 4.62 (1H, m, H-6), 4.49 (1H, m, H-4), 4.48 (1H, d, *J* = 8.0 Hz, H-1’), 3.86 (1H, d, *J* = 11.6 Hz, H-6’a), 3.66 (1H, dd, *J* = 11.6, 5.2 Hz, H-6’b), 3.22~3.40 (4H, m, H-2’~H-5’), 2.56 (1H, m, H-5a), 1.66 (1H, m, H-5b); ^13^C NMR (CD_3_OD, 100 MHz) *δ*: 159.1 (s, C-1), 143.3 (d, C-3), 124.5 (d, C-2), 117.9 (s, C-8), 102.6 (d, C-1’), 93.7 (d, C-7), 78.2 (d, C-5’), 78.0 (d, C-3’), 74.8 (d, C-6), 74.7 (d, C-2’), 71.5 (d, C-4’), 67.6 (d, C-4), 62.6 (t, C-6’), 36.4 (t, C-5).

### Serine protease inhibition

Enzyme inhibition assays are described in Supplementary information S2.

### Determination of O_2_^.−^ generation and elastase release from neutrophils

All assays were performed as described previously^58^,^59^. Neutrophils isolated from the blood of healthy volunteers (20–30 years old) were resuspended in a Ca^2+^-free HBSS buffer (pH 7.4) at 4 °C before use. O_2_^.−^ generation was measured based on reduction of ferricytochrome *c*. In brief, after supplementation with 0.5 mg/ml ferricytochrome *c* and 1 mM Ca^2+^, neutrophils were equilibrated at 37 °C for 2 min and incubated with the specified drugs for 5 min. Neutrophils were activated using 30 nM fMLF or 100 nM LTB4 for 10 min after addition of cytochalasin B (CB, 1 μg/mL) for 3 min. Changes in absorbance concomitant with reduction of ferricytochrome *c* at 550 nm were continuously monitored. For elastase release, neutrophils (6 × 10^5^ cells/mL) were mixed with methoxysuccinyl-Ala-Ala-Pro-Val-pNA (100 μM) substrate at 37 °C for 5 min. After incubation with DMSO or test agents, neutrophils were activated as described previously, and changes in absorbance at 405 nm continuously monitored to assay elastase release. Inhibition of superoxide generation and elastase release were calculated in keeping with previous reports.

### Immunoblotting assay

Neutrophils were pretreated with DMSO or 3,5-DCQA (10 μM) for 5 min before fMLF stimulation for 0.5 min at 37 °C. Cells were lysed with lysis buffer consisting of 50 mM HEPES (pH 7.4), 100 mM NaCl, 1 mM Ca^2+^, 2 mM Na_3_VO_4_, 1 mM phenylmethanesulfonyl fluoride, 5% b-mercaptoethanol, 10 mM p-nitrophenyl phosphate, 1% protease inhibitor cocktail (Sigma-Aldrich), and 1% Triton X-100. Cell lysates were collected by centrifugation at 14,000 rpm for 20 min at 4 °C. After gel electrophoresis and transferring to membranes, samples were blocked with 5% nonfat milk in a mixture of Tris-buffer saline and Tween 20. Target protein was identified by the corresponding primary antibody overnight at 4 °C. Membranes were incubated with horseradish peroxidase-conjugated, secondary anti-rabbit or anti-mouse antibodies at room temperature for 1 h. After washing, enhanced chemiluminescence solution was used and protein expression was analyzed by the BioSpectrum Imaging System (UVP, Upland, CA). The quantitative ratio of target protein was normalized to total protein or GAPDH.

### Animals

All animal experiments were performed in accordance with the guidelines of the Animal Welfare Act and The Guide for Care and Use of Laboratory Animals from the National Institutes of Health. The animal protocols were approved by the Institutional Animal Care and Use Committee of Chang Gung University (Taoyuan, Taiwan, IACUC Approval no.: CGU12-011, period of protocol valid from June 01, 2012 to May 31, 2015). Male ICR mice (5–6 weeks; 25–30 g) were purchased from BioLasco (Ilan, Taiwan). Mice were housed under standard laboratory conditions, and fed a standard laboratory diet and water *ad libitum*. Animals were allowed to adapt to the environment for at least one week before experiments.

### Acute oral toxicity

IKWE powder was dissolved in 600 μL ddH_2_O to generate a solution at a dose equivalent to 10.0 g/kg. Mice were administered IKWE solution (300 μL) or ddH_2_O via gavage at intervals of 4 h. After 24 h, blood (ca. 1.0 mL) was collected through cardiac puncture under anesthesia with intraperitoneal injection of Zoletil 50 (50 mg/kg) and Xylazine (10 mg/kg). Blood samples were immediately mixed with 100 μL acid citrate dextrose solution (BD Vacutainer, 364606), centrifuged at 1,000 rpm for 15 min and stored at −20 °C. Supernatant fractions were further analyzed to determine the AST, ALT, CRTN and BUN levels using FUJI DRI-CHEM 3000 and four FUJI DRI-CHEM SLIDE products (n = 10). LD_50_ and body weight growth were additionally determined for 14 days (n = 10)^23^. Data were presented as mean ± S.E.M.

### LPS-induced ALI in mice

Mice were randomly divided into nine groups (n = 6 per group): (1) Vehicle, (2) IKWE (500 mg/kg), (3) 3,5-DCQA (50 mg/kg), (4) LPS, (5) LPS + DEX (10 mg/kg), (6) LPS + IKWE (250 mg/kg), (7) LPS + IKWE (500 mg/kg), (8) LPS + 3,5-DCQA (25 mg/kg), and (9) LPS + 3,5-DCQA (50 mg/kg). Agents were dissolved in vehicle solution (PBS containing 10% Tween-80). All animals were pretreated intraperitoneally with 100 μL vehicle solution or agents, respectively, 1 h before PBS or LPS (Sigma, *Escherichia coli.* 055:B5) intratracheal injection (5 mg/kg in 50 μL of PBS)^26^,^27^. Mice were sacrificed at 6 h post-dosage, and the left lung collected for histological examination or MPO activity measurement. Lung tissue was fixed with 10% formalin before embedding with paraffin wax and routine H&E staining. MPO activity was measured as described earlier^59^. The right lobes were prepared for W/D weight ratio determination or BALF analysis. To obtain the W/D weight ratio, wet tissues were weighed immediately after collection, dried in the oven at 80 °C for 48 h, and re-weighed. For post-treatment experiments, mice treated with vehicle or 3,5-DCQA (50 mg/kg) intraperitoneally 1 h after LPS treatment^24^.

### Bronchoalveolar lavage fluid preparation and analysis

Mice were sacrificed and hilum of the left lung sealed. BALF was collected following injection with 0.2, 0.2, 0.3, 0.3 or 0.5 mL PBS. BALF samples were combined and centrifuged at 1,000 rpm for 15 min at 4 °C. Total proteins in the supernatant fraction were determined using the protein assay dye (BIO-RAD, 500-0006) with BSA as the reference, and TNF-α and IL-6 levels measured with ELISA kits (eBioscience, 88-7324-88 and 88-7064-88). Cell pellets from BALF were resuspended in 100 μL PBS and cytospun (200 g, 3 min) onto a glass microscope slide, followed by staining with Liu’s stain. Neutrophils were counted under a light microscope in five randomly selected fields (×200 magnification)^60^.

### Statistical analysis

All data were expressed as mean ± S.E.M. and analyzed with two-tailed indirect Student tests or one-way ANOVA followed by Dunnet’s multiple comparison test. GraphPad Prism 5.01 was applied for statistical analysis (GraphPad Software, Inc., USA).

## Additional Information

**How to cite this article**: Chen, Y.-L. *et al.* *Ilex kaushue* and Its Bioactive Component 3,5-Dicaffeoylquinic Acid Protected Mice from Lipopolysaccharide-Induced Acute Lung Injury. *Sci. Rep.* **6**, 34243; doi: 10.1038/srep34243 (2016).

## Supplementary Material

Supplementary Information

## Figures and Tables

**Table 1:** Serine proteases inhibition profiles of isolates and synthetics.

Compounds	IC_50_ (μM)				
	HNE	Cathepsin G	Proteinase 3	Thrombin	Chymotrypsin
**1**	>30	>30	>30	>30	>30
**2**	>30	>30	>30	>30	>30
**3**	>30	>30	>30	>30	>30
**4**	>30	>30	>30	>30	>30
**6**	1.86 ± 0.06	>30	>30	>30	>30
**7**	>30	>30	>30	>30	>30
**9**	11.52 ± 1.28	>30	7.33 ± 0.65	6.37 ± 0.60	6.06 ± 0.05
**10**	>30	>30	>30	>30	>30
**11**	>30	>30	>30	>30	>30
**12**	>30	>30	>30	>30	>30
**13**	>30	>30	>30	>30	>30
**14**	>30	>30	>30	>30	>30
**15**	>30	>30	>30	>30	>30
**17**	>30	>30	>30	>30	>30
**18**	>30	>30	>30	>30	>30
**19**	>30	>30	>30	>30	>30
**11-1**	>30	>30	7.62 ± 0.32	>30	8.06 ± 0.40
**11-2**	8.55 ± 1.77	>30	1.99 ± 0.25	2.98 ± 0.35	6.04 ± 0.29
**14-1**	>30	>30	>30	>30	>30
**15-1**	>30	>30	17.44 ± 0.16	24.94 ± 1.33	16.98 ± 0.10
**15-2**	24.75 ± 0.48	>30	14.77 ± 0.59	11.77 ± 2.20	11.40 ± 0.20
Elaspol^a^	0.03 ± 0.00	—	0.56 ± 0.06	—	—
CG inhibitor^a^	—	0.27 ± 0.03	—	—	—
AEBSF^a^	—	—	—	250.74 ± 8.78	30.96 ± 4.96

^a^Positive controls: Elaspol, CG inhibitor and AEBSF. Data were presented as mean ± S.E.M. (n ≥ 3).

**Table 2:** Inhibitory effects on fMLF/CB-activated neutrophils of isolates and synthetics.

Compounds	IC_50_ (μM)		Compounds	IC_50_ (μM)	
	O_2_^.−^ generation	HNE		O_2_^.−^ generation	HNE
**1**	1.54 ± 0.29	>10	**9**	0.99 ± 0.26	1.07 ± 0.33
**2**	2.81 ± 1.00	>10	**10**	>10	>10
**3**	2.56 ± 1.06	>10	**11**	>10	>10
**4**	1.70 ± 0.28	>10	**12**	>10	>10
**6**	1.92 ± 0.54	>10	**13**	>10	>10
**7**	1.49 ± 0.38	>10	**14**	>10	>10
QA	>10	>10	**15**	>10	7.99 ± 1.18
CA	1.42 ± 0.02	>10	**17**	>10	>10
RA	1.29 ± 0.09	>10	**18**	>10	>10
THBA	1.14 ± 0.08	>10	**19**	>10	>10
*p*-CA	>10	>10	**11**-**1**	>10	>10
3-HCA	>10	>10	**11**-**2**	>10	>10
FA	>10	>10	**14**-**1**	4.77 ± 0.40	2.84 ± 1.02
	—	—	**15**-**1**	0.14 ± 0.09	0.06 ± 0.01
	—	—	**15**-**2**	0.26 ± 0.10	0.20 ± 0.02

QA, quinic acid; CA, caffeic acid; RA, rosaminiric acid; THBA, 3,4,5-trihyroxybenzoic acid; *p*-CA, *p*-coumaric acid; 3-HCA, 3-hydroxycinnamic acid; FA, ferulic acid. Data were presented as mean ± S.E.M. (n ≥ 3).
