# Supplementary material for: Ilex kaushue and Its Bioactive Component 3,5-Dicaffeoylquinic Acid Protected Mice from Lipopolysaccharide-Induced Acute Lung Injury
Source: Sci Rep. 2016 Sep 29;6:34243. doi: 10.1038/srep34243 (PMC5041076; doi:10.1038/srep34243)
Supplement: Supplementary Information [file srep34243-s1.pdf]

Supplementary Information

***Ilex kaushue* and Its Bioactive Component 3,5-Dicaffeoylquinic Acid Protected Mice from Lipopolysaccharide-Induced Acute Lung Injury.**

Yu-Li Chen[1], Tsong-Long Hwang[1,2,3,4,5], Huang-Ping Yu[5,6], Jia-You Fang[1,2,3,4,5], Kowit Yu Chong[1,7], Yao-Wen Chang[1,2], Chun-Yu Chen[5], Hsuan-Wu Yang[2], Wen-Yi Chang[2] & Pei-Wen Hsieh[*1,2,4,5]

[1]Graduate Institute of Biomedical Sciences, College of Medicine, Chang Gung University, Taoyuan, Taiwan. [2]Graduate Institute of Natural Products, College of Medicine, Chang Gung University, Taoyuan, Taiwan. [3]Chinese Herbal Medicine Research Team, Healthy Aging Research Center, Chang Gung University, Taoyuan, Taiwan.[4] Research Center for Industry of Human Ecology and Research Center for Chinese Herbal Medicine, Chang Gung University of Science and Technology, Taoyuan, Taiwan. [5]Department of Anesthesiology, Chang Gung Memorial Hospital, Taoyuan, Taiwan. [6]School Medicine, College of Medicine, Chang Gung University, Taoyuan, Taiwan. [7]Department of Medical Biotechnology and Laboratory Science, College of Medicine, Chang Gung University, Taoyuan, Taiwan.

Correspondence and requests for materials should be addressed to P.-W.H. (pewehs@mail.cgu.edu.tw)

Supplementary information Figure S1
Supplementary information Figure S2
Supplementary information Figure S3
Supplementary information Figure S4
Supplementary information Figure S5
Supplementary information Figure S6
Supplementary information Figure S7
Supplementary information Figure S8
Supplementary information Figure S9
Supplementary information Figure S10
Supplementary information Figure S11
Supplementary information Figure S12
Supplementary information Table S1
Supplementary information Table S2
Supplementary information Table S3
Supplementary information S1
Supplementary information S2
Supplementary information Reference

Supplementary information Figure S1

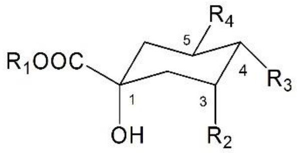

Caffeoyl group: 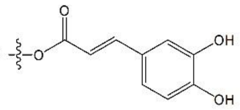

1: $R_1 = R_3 = R_4 = H$, R_2 = Caffeoyl group
2: $R_1 = R_2 = R_4 = H$, R_3 = Caffeoyl group
3: $R_1 = R_2 = R_3 = H$, R_4 = Caffeoyl group
4: $R_1 = R_4 = H$, $R_2 = R_3$ = Caffeoyl group
5: R_1 = Methyl group, $R_4 = H$, $R_2 = R_3$ = Caffeoyl group
6: $R_1 = R_3 = H$, $R_2 = R_4$ = Caffeoyl group
7: $R_1 = R_2 = H$, $R_3 = R_4$ = Caffeoyl group
8: R_1 = Methyl group, $R_2 = H$, $R_3 = R_4$ = Caffeoyl group

S1: 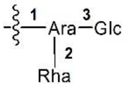

S2: 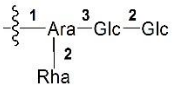

S3: 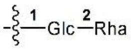

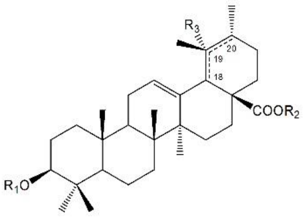

9: $R_1 = H$, $R_2 = H$, $R_3 = H$
15: $R_1 = S1$, $R_2 = S3$, $R_3 = OH$
15-1: $R_1 = H$, $R_2 = H$, C=C: C-18/C-19
15-2: $R_1 = H$, $R_2 = H$, C=C: C-19/C-20

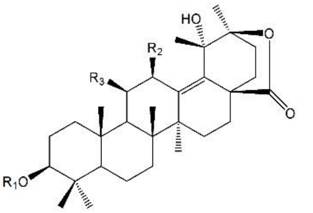

10: $R_1 = S1$, $R_2 = OH$, $R_3 = H$
12: $R_1 = S2$, $R_2 = OH$, $R_3 = H$
13: $R_1 = S1$, $R_2 = H$, $R_3 = OH$

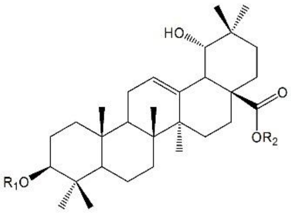

14: $R_1 = S1$, $R_2 = S3$
14-1: $R_1 = H$, $R_2 = H$

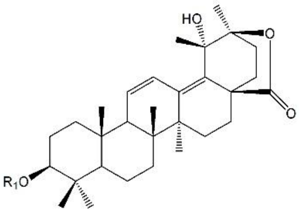

11: $R_1 = S1$
11-1: $R_1 = H$

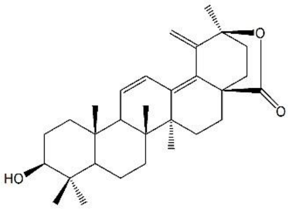

11-2

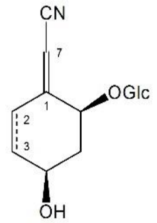

17: *Z* form: C-1/C-7
18: *Z* form: C-1/C-7, C=C: C-2/C-3
19: *E* form: C-1/C-7, C=C: C-2/C-3

Fig. S1. Structures of isolatic and semi-synthetic compounds

Supplementary infomration Figure S2

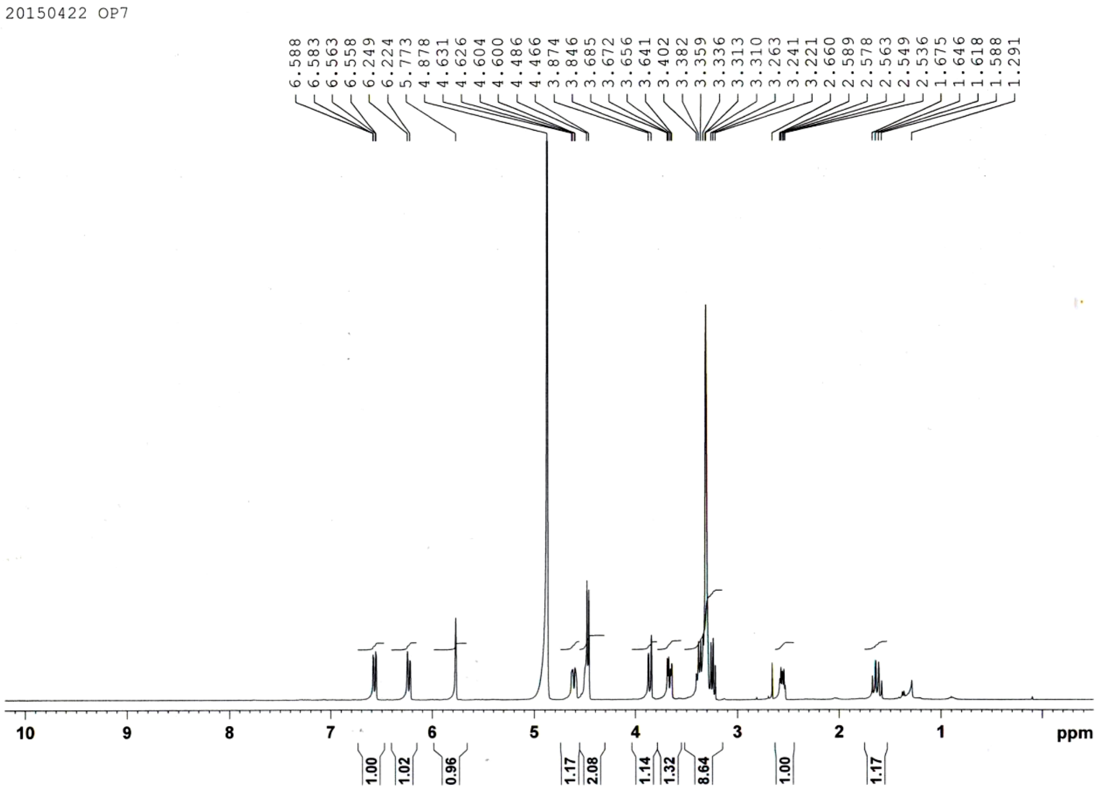

Fig. S2. ^{1}H NMR spectrum of menisdaurin F.

Supplementary infomration Figure S3

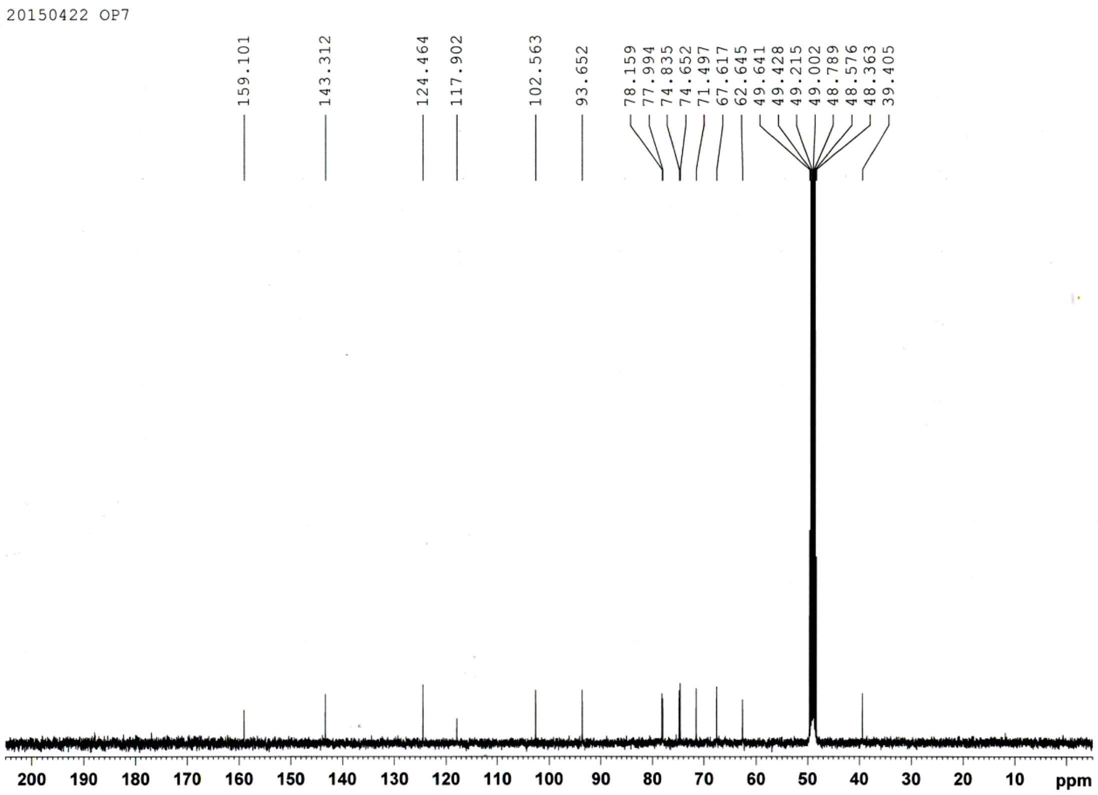

Fig. S3. ^{13}C NMR spectrum of menisdaurin F.

Supplementary infomration Figure S4

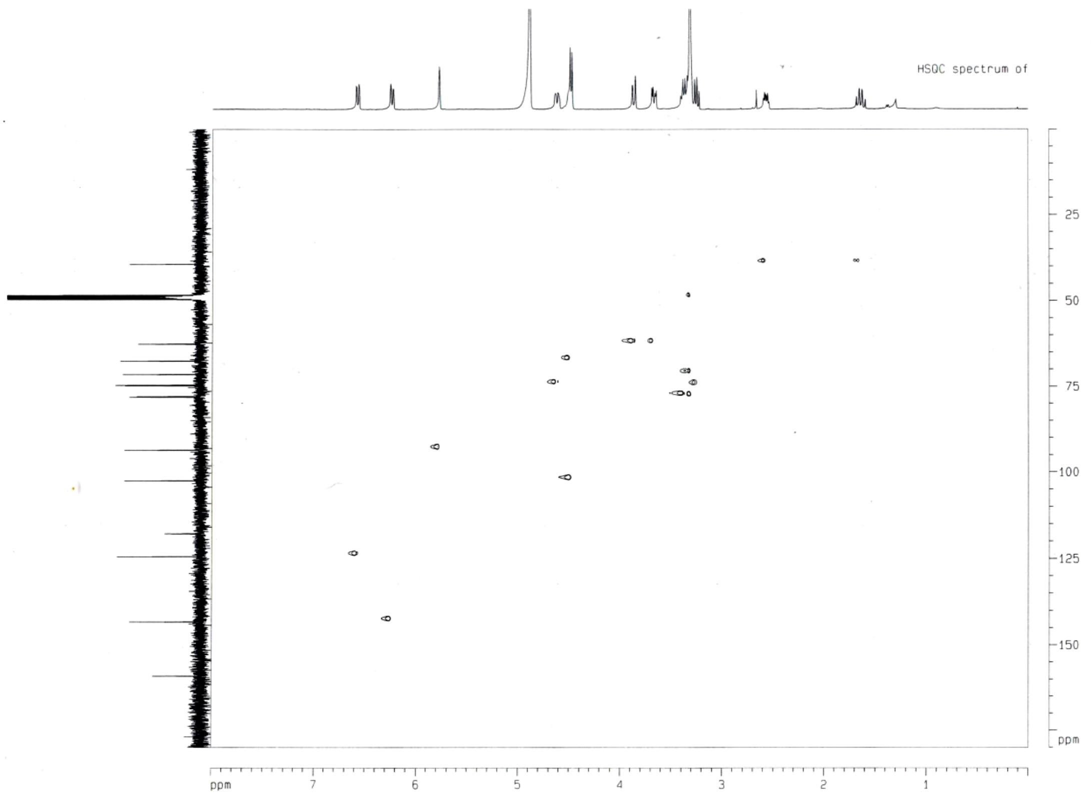

Fig. S4. HSQC spectrum of menisdaurin F.

Supplementary infomration Figure S5

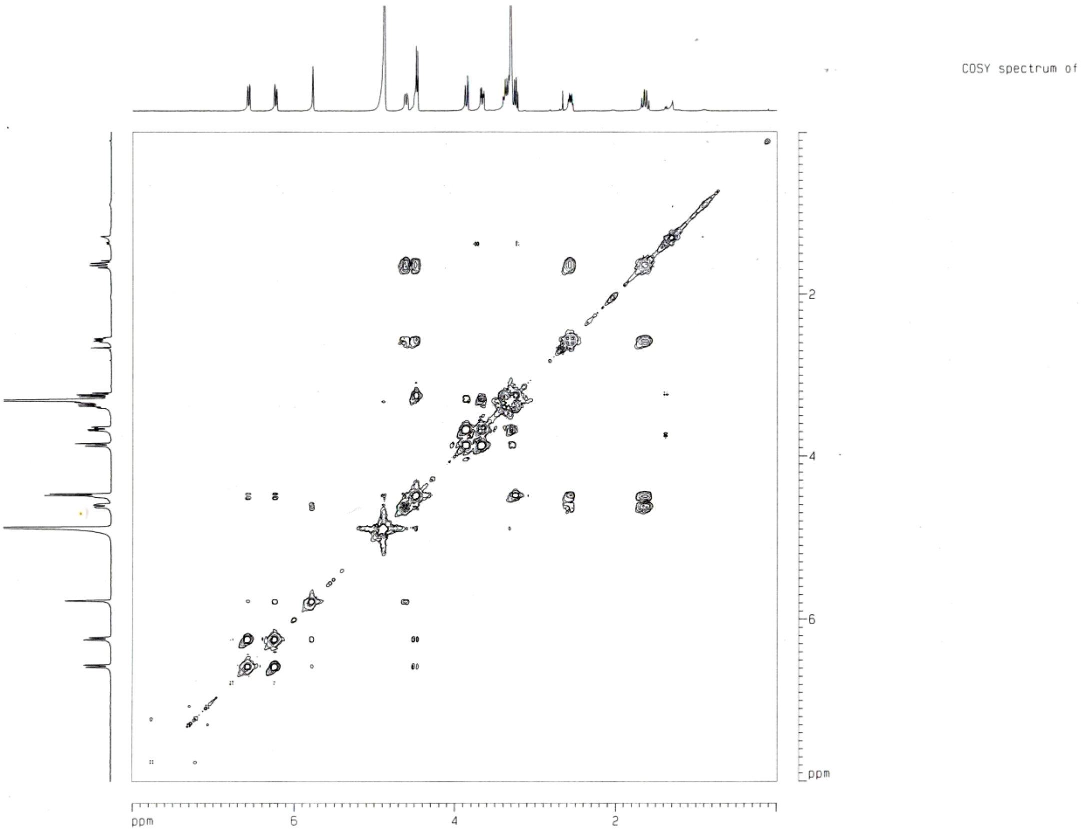

Fig. S5. COSY spectrum of menisdaurin F.

Supplementary infomration Figure S6

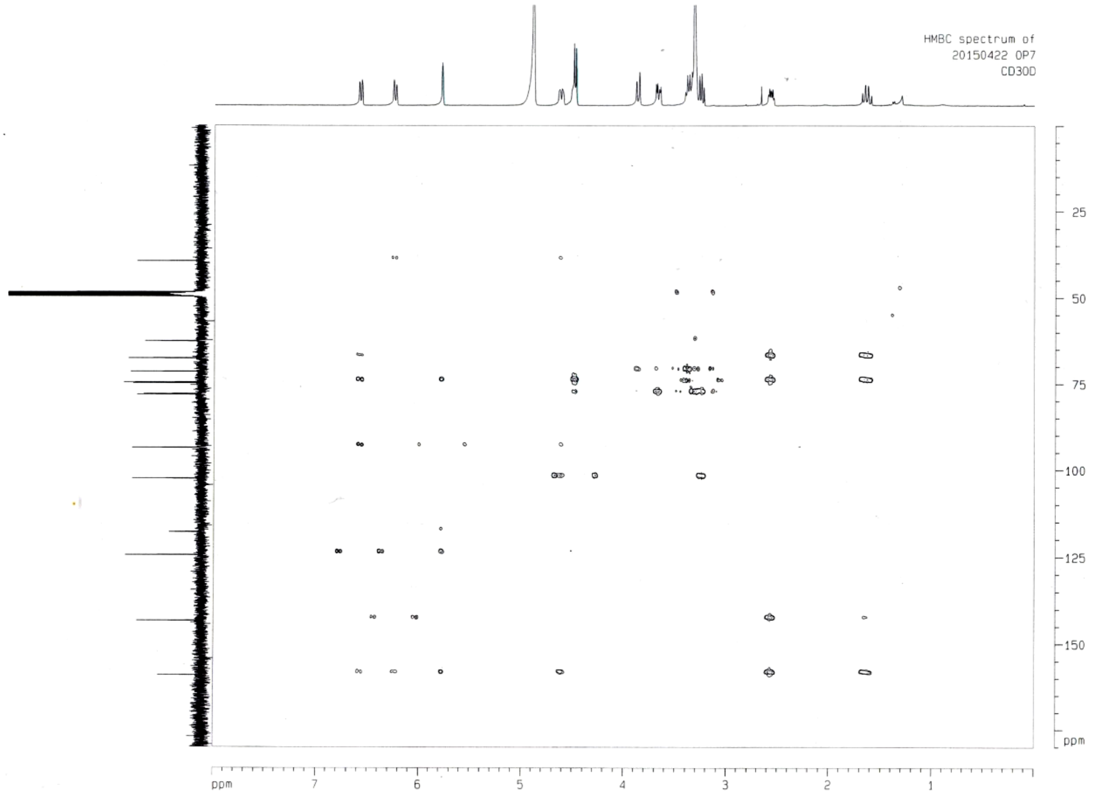

Fig. S6. HMBC spectrum of menisdaurin F.

Supplementary infomration Figure S7

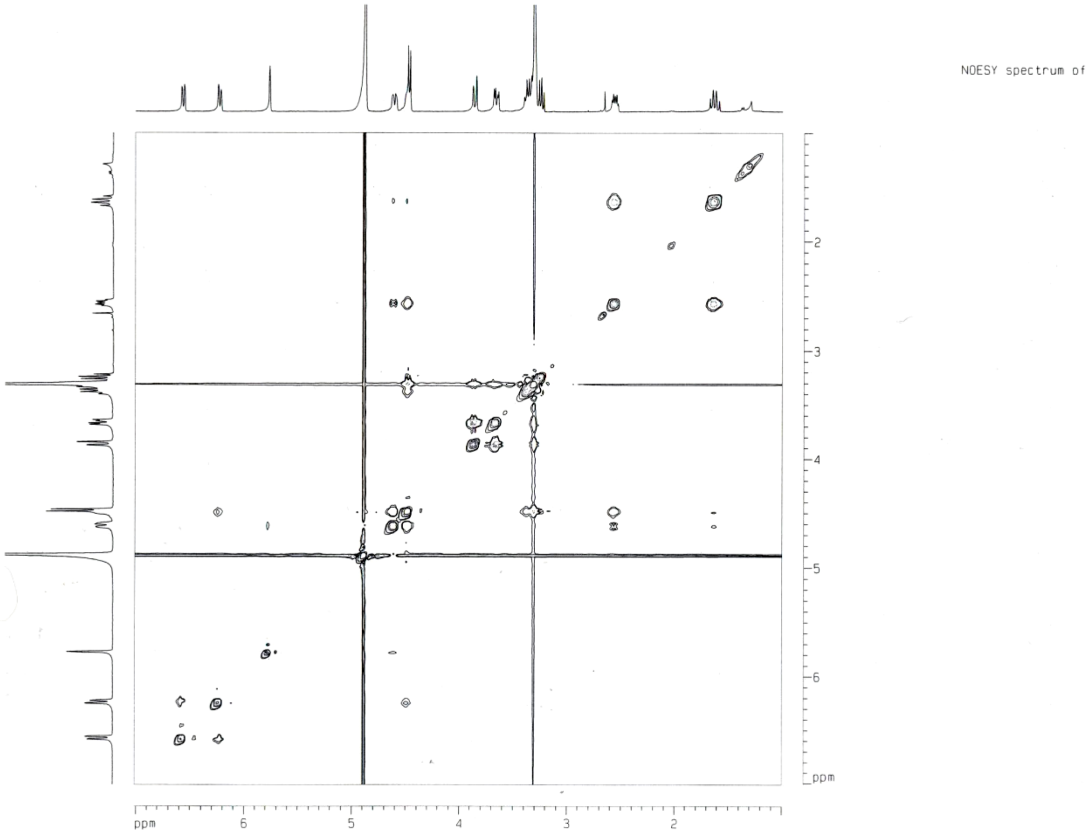

Fig. S7. NOESY spectrum of menisdaurin F.

Supplementary infomration Figure S8

D:\Exp_data\...\2015\12102015\32-OP7-H 12/10/2015 10:02:59 AM

32-OP7-H#1-20 RT: 0.01-0.42 AV: 20
T: FTMS + p ESI Full ms [150.00-2000.00]
m/z= 308.7158-354.8541

Isotope	Min	Max
N-14	0	1
O-16	0	7
C-12	0	14
H-1	0	25
Na-23	0	1

Charge 1
Mass tolerance 140.00 ppm
Nitrogen rule not used
RDB equiv -1.00-100.00
max results 1

m/z	Intensity	Relative	Theo. Mass	Delta (ppm)	Composition
336.1048	164920.2	100.00	336.1054	-1.85	$C_{14}H_{19}O_7NNa$

Fig. S8. HRESIMS spectrum of menisdaurin F.

Supplementary information Figure S9

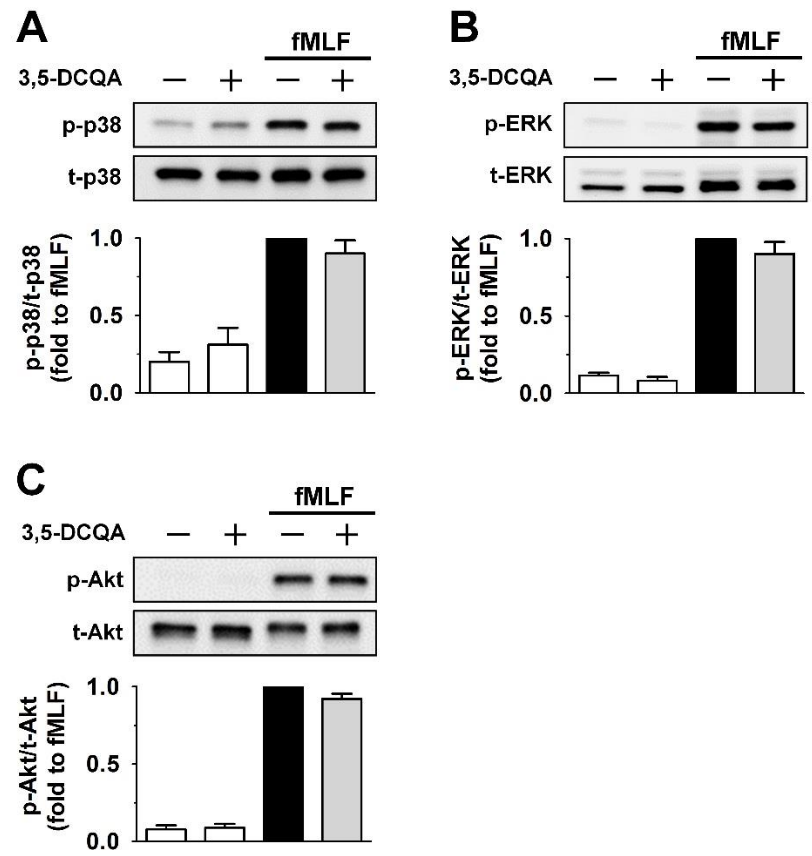

Fig. S9. 3,5-DCQA did not inhibited the phosphorylation of MAPKs and Akt in fMLF-activated human neutrohpils. Human neutrophils were pre-incubated with dimethylsulfoxide (DMSO) or 3,5-DCQA (10 μM) for 5 min before stimulation with or without fMLF (0.1 μM) for another 0.5 min. All the Western blotting experiments were performed under the same condition. The targeted proteins were immunoblotted with its specific monoclonal antibody. (**A**) p38; (**B**) ERK; (**C**) Akt. Targeted bands were analyzed using a densitometer and normalized to the corresponding total protein or glyceraldehyde 3-phosphate dehydrogenase (GAPDH). The densitometric data were presented as mean ± S.E.M. (n = 3–4). Compared with fMLF group: $^{\#}$ $p < 0.05$ and $^{\#\#}$ $p < 0.01$.

Supplementary information Figure S10

3,4-DCQA (**4**)

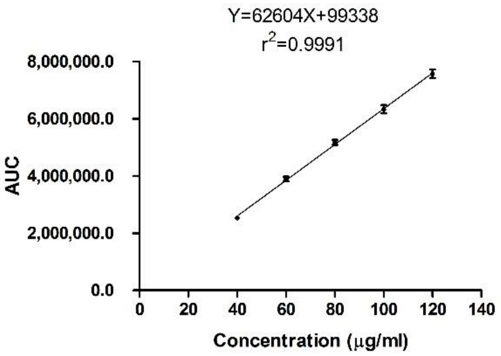

3,5-DCQA (**6**)

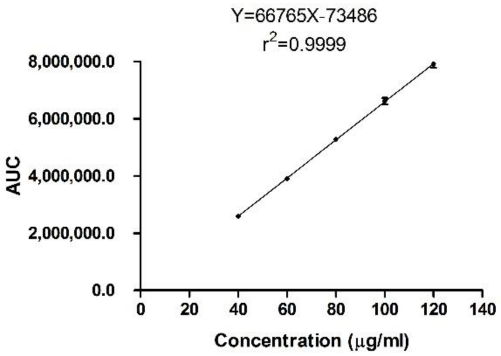

4,5-DCQA (**7**)

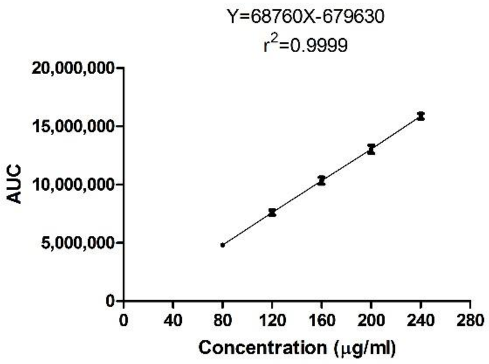

Fig. S10. Calibration curves of three chemical reference standards.

Supplementary infomration Figure S11

A

I. kaushue (IK) KUD NKUD

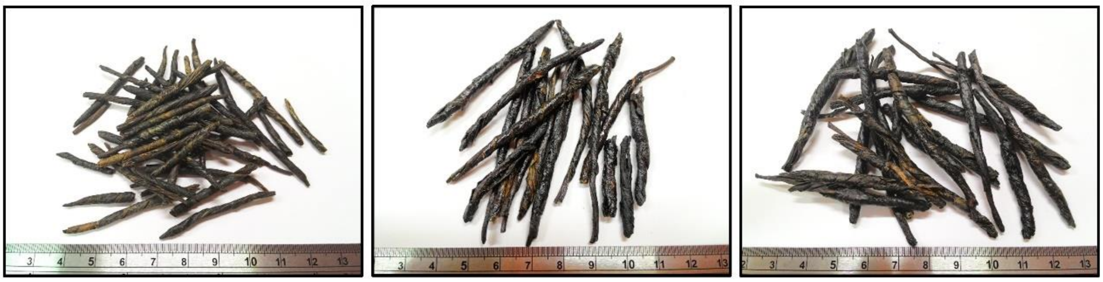

B

Batches	Sample (g)	Extracts (g)	Yield (%)	3,4-DCQA	3,5-DCQA	4,5-DCQA	HNE inhibition[a]
IKWE-1	20.0	7.33	36.65	3.70	4.78	9.09	11.37 ± 1.59
IKWE-2	21.1	7.96	37.74	3.34	4.45	8.58	10.40 ± 0.35
IKWE-3	20.8	8.41	40.43	4.25	4.87	8.89	9.73 ± 0.42
KUD-1	20.0	7.07	35.33	5.33	4.24	7.02	9.75 ± 1.09
NKUD-1	20.1	8.05	39.98	5.06	5.87	11.08	8.71 ± 0.15

Fig. S11. Quality control index of water extracts from different Kudingcha materials. (A) Three Kudincha materials; (B) Index of QC. [a]Concentration necessary for 50 % inhibition at μg/ml. Data were presented as mean ± S.E.M. (n ≥ 3).

Supplementary infomration Figure S12

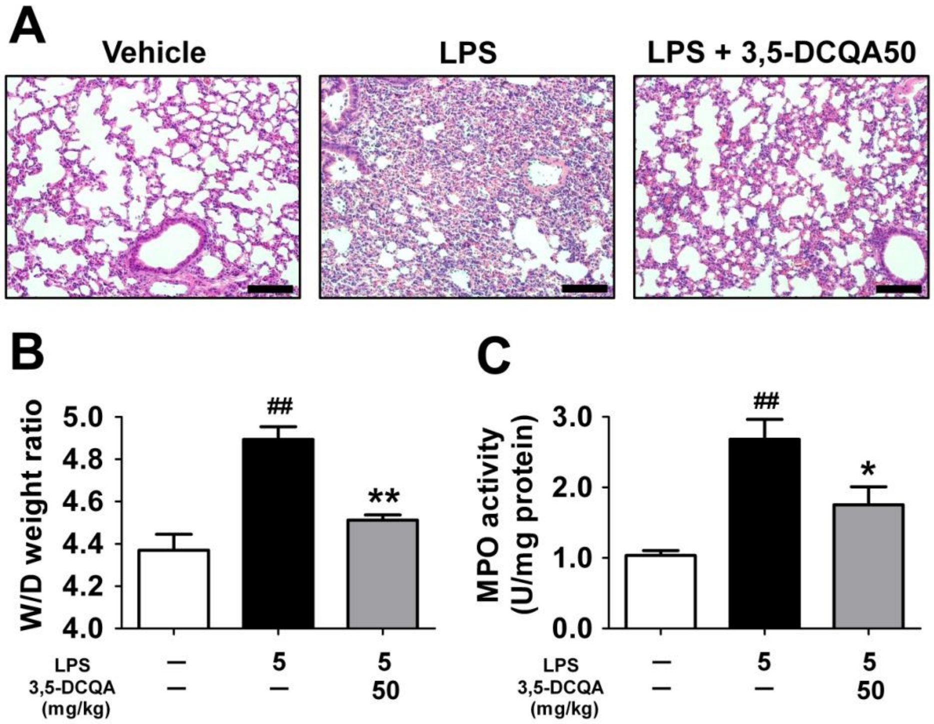

Fig. S12. The effects of post-treated 3,5-DCQA on LPS-induced ALI in mice. Mice received post-treatment of vehicle or 3,5-DCQA (50 mg/kg) intraperitoneally 1hour after intratracheal instillation of LPS. After 6 hours of LPS treatment, mice were anesthetized and their chests were opened. Whole lungs were obtained immediately. Left lobe was then dissected for histology or MPO activity. Right lobes were applied for W/D weight ratio. (**A**) Histological examination (scale bar = 50 μM); (**B**) (**C**) Lung W/D weight ratio and MPO activity. Data were presented as mean ± S.E.M. (n = 3 for control; n = 6 for LPS or 3,5-DCQA groups). Compared with vehicle group: ## $p < 0.01$; Compared with LPS group: * $p < 0.05$ and ** $p < 0.01$.

Supplementary information Table S1

Table S1. ^{1}H and ^{13}C NMR data of menisdaurin and menisdaurin F

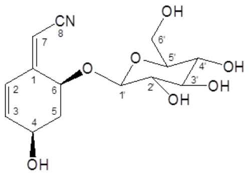

Mendisdaurin

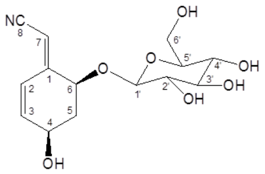

Mendisdaurin F

Position	Menisdaurine		Menisdaurine F	
	^{1}H	^{13}C	^{1}H	^{13}C
1		157.1, s		159.1, s
2	6.32, d, *J*=10.0 Hz	127.7, d	6.57, dd, *J*=10.0, 2.0 Hz	124.5, d
3	6.24, dd, *J*=10.0, 3.2 Hz	140.6, d	6.24, d, *J*=10.0 Hz	143.3, d
4	4.40, m	65.3, d	4.49, m	67.6, d
5	2.30, ddd, *J*=13.2, 5.2, 4.4 Hz<br>2.06, ddd, *J*=13.2, 8.0, 6.2 Hz	36.1, t	2.56, m<br>1.66, m	36.4, t
6	4.96, ddd, *J*=8.0, 2.4, 1.2 Hz	72.5, d	4.62, m	74.8, d
7	5.55, s	96.8, d	5.77, s	93.7, d
8		118.0, s		117.9, s
1'	4.58, d, *J*=6.4 Hz	101.5, d	4.48, d, *J*=8.0 Hz	102.6, d
2'	3.29~3.45, m	74.4, d	3.22~3.40, m	74.7, d
3'		77.9, d		78.0, d
4'		71.7, d		71.5, d
5'		78.1, d		78.2, d
6'	3.92, dd, *J*=11.6, 2.0 Hz<br>3.70, dd, *J*=11.6, 6.0 Hz	63.1, t	3.86, d, *J*=11.6 Hz<br>3.66, dd, *J*=11.6, 5.2 Hz	62.6, t

Supplementary information Table S2

Table S2. Effects of 3,5-DCQA on $O_2^{\bullet-}$ generation and elastase release in fMLF- or LTB4-induced human neutrophils

Inducers	IC_{50} (μM)	
	$O_2^{\bullet-}$ generation	elastase release
fMLF	1.92 ± 0.54	12.02 ± 0.60
LTB4	—	2.90 ± 0.55

All data were presented as mean ± S.E.M. (n ≥ 3).

Supplementary information Table S3

Table S3. HNE inhibitory activities of caffeic acid analogs

Compounds	IC_{50} (μM)	Inh %[a]
**1**	> 30	-2.99 ± 1.35
**2**	> 30	-3.43 ± 4.85
**3**	> 30	-4.76 ± 3.36
**4**	> 30	9.91 ± 0.99
**6**	1.86 ± 0.06	90.53 ± 0.52
**7**	> 30	36.42 ± 2.25
CA	> 30	-4.61 ± 4.03
p-CA	> 30	-4.00 ± 3.42
3-HCA	> 30	-3.63 ± 3.91
FA	> 30	0.06 ± 4.79
RA	> 30	10.58 ± 4.03

[a]HNE inhibition at 30 μM. CA, caffeic acid; *p*-CA, *p*-coumaric acid; 3-HCA, 3-hydroxycinnamic acid; FA, ferulic acid; RA, rosaminiric acid. All data were presented as mean ± S.E.M. (n ≥ 3).Data were presented as mean ± S.E.M. (n ≥ 3).

Supplementary information S1

General Experimental Procedures

Thin-layer chromatography (TLC) was used Merck 60 F254 silica gel aluminum backed plates. Flash column chromatography was performed using silica gel (Silicycle, 70-230 mesh or 230-400 mesh). The HPLC system was performed using Jasco PU-1580 intelligent HPLC pump, Jasco AS 1555-10 intelligent sampler, and Jassco UV-1575 UV-Vis detector. Semi-preparative (Develosil, C30-UG-5, 10 mm × 250 mm) or preparative column (Shiseido, CAPCELL PAK C18 AQ, 20 mm × 250 mm) were applied for compound purification. The MPLC-flash system was performed using Biotage® Isolera One with SNAP® KP-C18-HS 60 g cartridge column, and detection was conducted at 254 and 330 nm. The nuclear magnetic resonance (NMR) spectra using CD_3OD, DMSO-d_6 and C_5D_5N as the solvent were obtained on a Bruker AVANCE-400 MHz FT-NMR spectrometer. Low-resolution EI-MS were recorded on a Quattro GC/MS spectrometer having a direct inlet system, low-resolution and high-resolution ESI-MS spectra on a Bruker Daltonics APEX II 30e spectrometer.

Bioactivity-guided fractionation

IKWE (5.50 g) was dissolved in ddH_2O and partitioned with ethyl acetate and *n*-butanol sequentially to generate the ethyl acetate (EA) (281.3 mg), *n*-BuOH (2.6 g), and H_2O (2.2 g) layers, respectively. The HNE activity assay was employed to monitor sub-fraction bioactivity. Ursolic acid (**9**) (26.6 mg) and β-sitosterol (**16**) (6.3 mg) were purified from the EA layer through repeated silica gel chromatography with the $CHCl_3$/MeOH (40/1, v/v) elution system.[1,2] The *n*-BuOH layer (2.0 g) was divided into five fractions (Bu-1, 175.7 mg; Bu-2, 127.3 mg; Bu-3, 296.5 mg; Bu-4, 420.3 mg; Bu-5, 720.7 mg) using the gradient MPLC-Flash system (1500 mL H_2O/MeOH gradient elution from 100% H_2O to 100% MeOH). Menisdaurin F (**19**) (7.3 mg; rt, 44.8 min), menisdaurin D (**17**) (31.3 mg; rt, 51.2 min) and menisdaurin (**18**) (36.5 mg; rt, 56.1 min) were derived from fraction Bu-2 with 18% MeOH aqueous solution at 3 mL/min.[3-6] Bu-4 was further divided into 3 fractions (Bu-4-1, 55.0 mg; Bu-4-2, 33.8 mg; Bu-4-3, 136.2 mg) with mobile phase consisting of 1% formic acid in 48% MeOH solution. 3,4-DCQA (**4**) (38.5 mg; rt, 72.0 min) and methyl 3,4-DCQA (**5**) (5.3 mg; rt, 68.8 min) were purified from Bu-4-1 with 1% formic acid in 40% MeOH solution at 3 mL/min.[7,8] 3,5-DCQA (**6**) (26.0 mg; rt, 49.2 min) was obtained from Bu-4-2 with 1% formic acid in 44% MeOH solution at 3.5 mL/min.[8] 4,5-DCQA (**7**) (55.3 mg; rt, 89.2 min) and methyl 4,5-DCQA (**8**) (11.8 mg; rt, 83.3 min) were

purified from Bu-4-3 with 1% formic acid in 43% MeOH solution at 3.0 mL/min.[7,8] Bu-5 was divided into two fractions (Bu-5-1, 480.6 mg; Bu-5-2, 135.2 mg) with 80% MeOH solution. Bu-5-1 was separated to give latifolioside H (**14**) (31.6mg; rt, 84.0 min) and latifolioside G (**15**) (85.0 mg; rt, 86.8 min) with 59% MeOH solution at 3 mL/min.[9] Bu-5-2 was separated to provide kudinoside C (**12**) (27.0 mg; rt, 38.8 min), kudinoside A (**10**) (45.0 mg; 42.8 min), kudinoside D (**11**) (11.5 mg; 49.6 min) and kudinoside F (**13**) (20.6 mg; 51.7 min) with 75% MeOH solution at 3.5 mL/min.[10-13] 3-CQA (**1**) (26.7 mg; rt, 9.1 min), 5-CQA (**3**) (57.3 mg; rt, 16.6 min) and 4-CQA (**2**) (33.1 mg; rt, 23.3 min) were purified directly with 25% MeOH mobile phase and 1% formic acid at 3.0 mL/min from IKWE solution (1.6 g in 20 mL of 25% MeOH solution).[8,14,15] For acid hydrolysis, 30 mg of compounds **11**, **14** and **15** were heated in 2 mL of 1 M HCl (H_2O : *p*-dioxane = 1 : 1) solution at 95℃ for 30 min. Reaction mixtures were partitioned with EA/H_2O three times. Aglycons were obtained from concentrated EA layers via HPLC purification: α-kudinlactone (**11-1**) (2.3 mg; rt, 7.6 min; 80% MeOH; 2.5 mL/min); kudinchagenin I (**11-2**) (1.5 mg; rt 16.1 min; 80% MeOH; 2.5 mL/min); siaresinolic acid (**14-1**) (2.7 mg; rt, 10.1 min; 90% MeOH; 2.5 mL/min); randialic acid B (**15-1**) (2.8 mg; rt, 22.3 min; 90% MeOH; 2.5 mL/min); sanguisorbigenin (**15-2**) (2.8 mg; rt, 25.4 min; 90% MeOH; 2.5 mL/min).[9,11-13] Compound structures were determined based on 1D/2D NMR and physical properties,

in comparison with reference data.

Neochlorogenic acid (**1**)

Light green powder; $[\alpha]^{22}_{D}$: -51.7° (c = 0.05, MeOH); UV λ_{max} (MeOH) nm (logε): 207 (3.92), 218 (3.98), 244 (3.83), 295 (3.91), 328 (4.02); IR ν_{max} (KBr) cm^{-1}: 3421, 1697, 1606, 1523, 1446, 983; Mp: 202-203°; ESIMS: m/z 353.1 $[M-H]^{+}$; ^{1}H NMR (CD_3OD, 400 MHz) δ: 7.58 (1H, d, J = 16.0 Hz, H-3'), 7.05 (1H, d, J = 2.0 Hz, H-5'), 6.94 (1H, dd, J = 8.4, 2.0 Hz, H-9'), 6.78 (1H, d, J = 8.4 Hz, H-8'), 6.31 (1H, d, J = 16.0 Hz, H-2'), 5.37 (1H, m, H-3), 4.17 (1H, m, H-5), 3.66 (1H, dd, J = 8.4, 3.2 Hz, H-4), 2.14~2.19 (3H, m, H-2a, H-6a, H-6b), 1.97 (1H, m, H-2b); ^{13}C NMR (CD_3OD, 100 MHz) δ: 178.3 (s, C-7), 169.0 (s, C-1'), 149.3 (s, C-7'), 146.8 (d, C-3'), 146.7 (s, C-6'), 127.9 (s, C-4'), 122.9 (d, C-9'), 116.5 (d, C-8'), 115.8 (d, C-2'), 115.1 (d, C-5'), 75.4 (s, C-1), 74.8 (d, C-4), 73.0 (d, C-3), 68.2 (d, C-5), 41.5 (t, C-6), 36.7 (t, C-2).

Cryptochlorogenic acid (**2**)

Light green powder; $[\alpha]^{22}_{D}$: -82.0° (c = 0.05, MeOH); UV λ_{max} (MeOH) nm (log ε): 207 (4.09), 218 (3.98), 244 (3.97), 295 (4.10), 328 (4.19); IR ν_{max} (KBr) cm^{-1}: 3414, 1695, 1606, 1520, 1447, 978; Mp: 205-206°; ESIMS: m/z 353.3 $[M-H]^{+}$; ^{1}H NMR (CD_3OD, 400 MHz) δ: 7.64 (1H, d, J = 16.0 Hz, H-3'), 7.07 (1H, d, J = 1.6 Hz, H-5'),

6.96 (1H, dd, J = 8.0, 1.6 Hz, H-9'), 6.79 (1H, d, J = 8.0 Hz, H-8'), 6.37 (1H, d, J = 16.0 Hz, H-2'), 4.81 (1H, dd, J = 9.2, 2.4 Hz, H-4), 4.32 (2H, m, H-3, H-5), 2.02~2.24 (4H, m, H-2a, H-2b, H-6a, H-6b). ^{13}C NMR (CD_3OD, 100 MHz) δ: 177.3 (s, C-7), 169.0 (s, C-1'), 149.4 (s, C-7'), 147.1 (d, C-3'), 146.7 (s, C-6'), 127.8 (s, C-4'), 123.0 (d, C-9'), 116.5 (d, C-8'), 115.2 (d, C-2'), 115.3 (d, C-5'), 79.2 (d, C-4), 76.6 (s, C-1), 69.6 (d, C-3), 65.5 (d, C-5), 42.6 (t, C-6), 38.4 (t, C-2).

Chlorogenic acid (**3**)

Light green powder; $[\alpha]^{22}_{D}$: -60.3° (c = 0.05, MeOH); UV λ_{max} (MeOH) nm (log ε): 206 (3.86), 218 (4.28), 244 (4.12), 297 (4.18), 328 (4.29); IR ν_{max} (KBr) cm^{-1}: 3409, 1697, 1604, 1521, 1447, 979; Mp: 210-202°; ESIMS: m/z 353.3 $[M-H]^+$; ^{1}H NMR (CD_3OD, 400 MHz) δ: 7.56 (1H, d, J = 16.0 Hz, H-3'), 7.06 (1H, s, H-5'), 6.95 (1H, d, J = 8.0 Hz, H-9'), 6.79 (1H, d, J = 8.0 Hz, H-8'), 6.26 (1H, d, J = 16.0 Hz, H-2'), 5.35 (1H, m, H-5), 4.19 (1H, m, H-3), 3.75 (1H, dd, J = 8.4, 2.4 Hz, H-4), 2.04~2.27 (4H, m, H-2a, H-2b, H-6a, H-6b); ^{13}C NMR (CD_3OD, 100 MHz) δ: 177.0 (s, C-7), 168.7 (s, C-1'), 149.3 (s, C-7'), 147.0 (d, C-3'), 146.5 (s, C-6'), 127.7 (s, C-4'), 123.0 (d, C-9'), 116.5 (d, C-8'), 115.2 (d, C-2'), 115.21 (d, C-5'), 76.1 (s, C-1), 73.5 (d, C-5), 71.8 (d, C-4), 71.3 (d, C-3), 38.8 (t, C-2), 38.1 (t, C-6).

3,4-Dicaffeoylquinic acid (**4**)

Light green powder; $[\alpha]^{22}_{D}$: -220.0° (c = 0.05, MeOH); UV λ_{max} (MeOH) nm (log ε): 218 (4.56), 244 (4.40), 300 (4.53), 328 (4.62); IR ν_{max} (KBr) cm^{-1}: 3382, 1694, 1603, 1521, 1446, 979; Mp: 153-154°; ESIMS: m/z 515.2 $[M-H]^+$; ^1H NMR (CD_3OD, 400 MHz) δ: 7.58 (1H, d, J = 16.0 Hz, H-3'), 7.55 (1H, d, J = 16.0 Hz, H-3''), 7.04 (1H, d, J = 1.2 Hz, H-5'), 7.02 (1H, d, J = 1.2 Hz, H-5''), 6.92 (1H, dd, J = 8.0, 1.2 Hz, H-9'), 6.87 (1H, dd, J = 8.0, 1.2 Hz, H-9''), 6.77 (1H, d, J = 8.0 Hz, H-8'), 6.73 (1H, d, J = 8.0 Hz, H-8''), 6.29 (1H, d, J = 16.0 Hz, H-2''), 6.25 (1H, d, J = 16.0 Hz, H-2'), 5.65 (1H, m, H-3), 5.01 (1H, dd, J = 8.8, 2.8 Hz, H-4), 4.39 (1H, m, H-5), 2.14~2.39 (4H, m, H-2a, H-2b, H-6a, H-6b); ^{13}C NMR (CD_3OD, 100 MHz) δ: 178.0 (s, C-7), 168.6 (s, C-1'), 168.5 (s, C-1''), 149.3 (s, C-7'), 149.3 (s, C-7''), 147.3 (d, C-3'), 147.3 (d, C-3''), 146.6 (s, C-6'), 146.6 (s, C-6''), 127.7 (s, C-4''), 127.6 (s, C-4'), 123.2 (d, C-9''), 123.1 (s, C-9'), 116.5 (d, C-8''), 116.4 (s, C-8'), 115.2 (d, C-5'), 115.1 (s, C-5''), 115.0 (d, C-2'), 114.8 (d, C-2''), 76.4 (d, C-4), 75.2 (s, C-1), 70.1 (d, C-3), 65.8 (d, C-5), 41.8 (t, C-6), 36.9 (t, C-2).

Methyl 3,4-Dicaffeoylquinic acid (**5**)

Light green powder; $[\alpha]^{22}_{D}$: -240.4° (c = 0.05, MeOH); UV λ_{max} (MeOH) nm (log ε): 207 (4.37), 218 (4.45), 244 (4.30), 296 (4.42), 328 (4.54); IR ν_{max} (KBr) cm^{-1}: 3392,

1691, 1604, 1521, 1446, 978; Mp: 159-160°; ESIMS: *m*/*z* 529.2 $[M-H]^+$; ^1H NMR (CD_3OD, 400 MHz) δ: 7.56 (1H, d, J = 16.0 Hz, H-3'), 7.55 (1H, d, J = 16.0 Hz, H-3''), 7.03 (1H, d, J = 2.0 Hz, H-5'), 7.02 (1H, d, J = 2.0 Hz, H-5''), 6.91 (1H, dd, J = 8.4, 2.0 Hz, H-9'), 6.89 (1H, dd, J = 8.4, 2.0 Hz, H-9''), 6.76 (1H, d, J = 8.0 Hz, H-8'), 6.73 (1H, d, J = 8.0 Hz, H-8''), 6.27 (2H, d, J = 16.0 Hz, H-2', H-2''), 5.62 (1H, m, H-3), 5.03 (1H, dd, J = 8.4, 3.2 Hz, H-4), 4.31 (1H, m, H-5), 3.71 (s, -COO$\underline{C}$H3), 2.10~2.38 (4H, m, H-2a, H-2b, H-6a, H-6b); ^{13}C NMR (CD_3OD, 100 MHz) δ: 176.1 (s, C-7), 168.5 (s, C-1'), 168.5 (s, C-1''), 149.6 (s, C-7'), 149.6 (s, C-7''), 147.4 (d, C-3'), 147.4 (d, C-3''), 146.8 (s, C-6'), 146.8 (s, C-6''), 127.8 (s, C-4''), 127.7 (s, C-4'), 123.2 (d, C-9''), 123.1 (s, C-9'), 116.5 (d, C-8'), 116.5 (s, C-8''), 115.2 (d, C-5'), 115.2 (s, C-5''), 115.1 (d, C-2'), 114.9 (d, C-2''), 75.5 (d, C-4), 75.2 (s, C-1), 69.8 (d, C-3), 66.0 (d, C-5), 52.9 (q, -COO$\underline{C}H_3$), 41.3 (t, C-6), 36.8 (t, C-2).

3,5-Dicaffoylquinic acid (**6**)

Light green powder; $[\alpha]^{22}_D$: -191.9° (c = 0.05, MeOH); UV λ_{max} (MeOH) nm (log ε): 207 (4.30), 218 (4.39), 244 (4.24), 297 (4.37), 328 (4.50); IR ν_{max} (KBr) cm^{-1}: 3319, 1692, 1603, 1523, 1446, 977; Mp: 163-164°; ESIMS: *m*/*z* 515.2 $[M-H]^+$; ^1H NMR (CD_3OD, 400 MHz) δ: 7.62 (1H, d, J = 16.0 Hz, H-3'), 7.58 (1H, d, J = 16.0 Hz, H-3''), 7.07 (2H, s, H-5', H-5''), 6.98 (1H, dd, J = 8.4, 2.0 Hz, H-9'), 6.96 (1H, dd, J =

8.4, 2.0 Hz, H-9''), 6.78 (2H, d, J = 8.4 Hz, H-8', H-8''), 6.36 (1H, d, J = 16.0 Hz, H-2''), 6.27 (1H, d, J = 16.0 Hz, H-2'), 5.43 (1H, m, H-3), 5.40 (1H, m, H-5), 3.98 (1H, dd, J = 7.6, 3.2 Hz, H-4), 2.13~2.34 (4H, m, H-2a, H-2b, H-6a, H-6b); ^{13}C NMR (CD_3OD, 100 MHz) δ: 177.5 (s, C-7), 168.9 (s, C-1'), 168.3 (s, C-1''), 149.6 (s, C-7'), 149.5 (s, C-7''), 147.3 (d, C-3'), 147.0 (d, C-3''), 146.8 (s, C-6'), 146.8 (s, C-6''), 127.9 (s, C-4'), 127.8 (s, C-4'), 123.1 (d, C-9'), 123.0 (s, C-9''), 116.4 (d, C-8'), 116.5 (s, C-8''), 115.6 (d, C-5'), 115.2 (s, C-5''), 115.1 (d, C-2'), 115.1 (d, C-2''), 74.7 (s, C-1), 72.5 (d, C-3), 72.1 (d, C-5), 70.6 (d, C-4), 37.7 (t, C-6), 36.0 (t, C-2).

4,5-Dicaffeoylquinic acid (**7**)

Light green powder; $[\alpha]^{22}_{D}$: -170.4° (c = 0.05, MeOH); UV λ_{max} (MeOH) nm (log ε): 207 (4.37), 218 (4.43), 246 (4.28), 297 (4.40), 328 (4.51); IR ν_{max} (KBr) cm^{-1}: 3369, 1694, 1604, 1523, 1446, 980; Mp: 158-159°; ESIMS: m/z 515.2 $[M-H]^{+}$; ^{1}H NMR (CD_3OD, 400 MHz) δ: 7.60 (1H, d, J = 16.0 Hz, H-3'), 7.52 (1H, d, J = 16.0 Hz, H-3''), 7.03 (1H, s, H-5'), 7.01 (1H, s, H-5''), 6.92 (1H, d, J = 7.6 Hz, H-9'), 6.90 (1H, d, J = 7.6 Hz, H-9''), 6.75 (2H, d, J = 7.6 Hz, H-8', H-8''), 6.29 (1H, d, J = 16.0 Hz, H-2''), 6.21 (1H, d, J = 16.0 Hz, H-2'), 5.63 (1H, m, H-5), 5.13 (1H, d, J = 7.2 Hz, H-4), 4.38 (1H, m, H-3), 2.03~2.27 (4H, m, H-2a, H-2b, H-6a, H-6b); ^{13}C NMR (CD_3OD, 100 MHz) δ: 176.8 (s, C-7), 168.5 (s, C-1'), 168.2 (s, C-1''), 149.6 (s, C-7'),

149.6 (s, C-7''), 147.7 (d, C-3'), 147.6 (d, C-3''), 146.7 (s, C-6'), 146.7 (s, C-6''), 127.6 (s, C-4'), 127.6 (s, C-4''), 123.2 (d, C-9'), 123.2 (s, C-9''), 116.4 (d, C-8'), 116.4 (s, C-8''), 115.1 (d, C-5'), 115.1 (s, C-5''), 114.7 (d, C-2'), 114.6 (d, C-2''), 76.0 (d, C-4), 75.6 (s, C-1), 69.3 (d, C-3), 69.0 (d, C-5), 39.3 (t, C-6), 38.3 (t, C-2).

Methyl 4,5-Dicaffeoylquinic acid (**8**)

Light green powder; $[\alpha]^{22}_{D}$: -162.7° (c = 0.05, MeOH); UV λ_{max} (MeOH) nm (log ε): 206 (4.32), 218 (4.41), 245 (4.27), 298 (4.39), 329 (4.52); IR ν_{max} (KBr) cm^{-1}: 3424, 1693, 1604, 1522, 1445, 982; Mp: 156-157°; ESIMS: m/z 529.3 $[M-H]^+$; ^1H NMR (CD_3OD, 400 MHz) δ: 7.61 (1H, d, J = 16.0 Hz, H-3'), 7.51 (1H, d, J = 16.0 Hz, H-3''), 7.04 (1H, d, J = 2.0 Hz, H-5'), 7.01 (1H, d, J = 2.0 Hz, H-5''), 6.94 (H, dd, J = 8.0, 2.0 Hz, H-9'), 6.92 (1H, dd, J = 8.0, 2.0 Hz, H-9''), 6.76 (2H, d, J = 8.0 Hz, H-8', H-8''), 6.30 (1H, d, J = 16.0 Hz, H-2''), 6.18 (1H, d, J = 16.0 Hz, H-2'), 5.53 (1H, m, H-5), 5.11 (1H, dd, J = 8.0, 2.8 Hz, H-4), 4.35 (1H, m, H-3), 2.34 (3H, s, -COO$\underline{C}H_3$), 2.07~2.34 (4H, m, H-2a, H-2b, H-6a, H-6b); ^{13}C NMR (CD_3OD, 100 MHz) δ: 175.2 (s, C-7), 168.5 (s, C-1'), 167.9 (s, C-1''), 149.8 (s, C-7'), 149.7 (s, C-7''), 147.7 (d, C-3'), 147.7 (d, C-3''), 146.8 (s, C-6'), 146.8 (s, C-6''), 127.7 (s, C-4'), 127.5 (s, C-4''), 123.2 (d, C-9'), 123.1 (s, C-9''), 116.5 (d, C-8'), 116.5 (s, C-8''), 115.1 (d, C-5'), 115.1 (s, C-5''), 114.7 (d, C-2'), 114.5 (d, C-2''), 75.7 (d, C-4), 74.7 (s, C-1), 69.1 (d, C-3),

68.5 (d, C-5), 53.1 (q, -COOCH3), 38.4 (t, C-2), 38.4 (t, C-6).

Ursolic acid (**9**)

White powder; $[\alpha]^{22}_{D}$: 87.4° (c = 0.05, MeOH); UV λ_{max} (MeOH) nm (log ε): 207 (3.59); IR ν_{max} (KBr) cm^{-1}: 3421, 2927, 2856, 1691, 1459, 1387, 1091; Mp: 276-277°; R_f = 0.37 ($CHCl_3$: MeOH = 40 : 1, v/v); ESIMS: m/z 455.4 $[M-H]^+$; ^1H NMR (C_5D_5N, 400 MHz) δ: 5.50 (1H, br s, H-12), 3.46 (1H, dd, J =9.6, 6.0 Hz, H-3), 2.64 (1H, d, J =11.2 Hz, H-18), 1.25 (3H, s, H-23), 1.24 (3H, s, H-27), 1.06 (3H, s, H-26), 1.03 (3H, s, H-24), 1.02 (3H, d, J = 6.4 Hz, H-29), 0.97 (3H, d, J = 5.6 Hz, H-30); ^{13}C NMR (C_5D_5N, 100 MHz) δ: 179.9 (s, C-28), 139.3 (s, C-13), 125.6 (d, C-12), 78.1 (d, C-3), 55.8 (d, C-5), 53.5 (d, C-18), 48.0 (d, C-9), 48.0 (s, C-17), 42.5 (s, C-14), 40.0 (s, C-8), 39.5 (d, C-20), 39.4 (s, C-4), 39.4 (d, C-19), 39.1 (t, C-1), 37.4 (t, C-22), 37.3 (s, C-10), 33.6 (t, C-7), 31.1 (t, C-21), 28.8 (q, C-23), 28.7 (t, C-15), 28.1 (t, C-2), 24.9 (t, C-16), 23.9 (q, C-27), 23.6 (t, C-11), 21.4 (q, C-30), 18.8 (t, C-6), 17.5 (q, C-29), 17.4 (q, C-26), 16.6 (q, C-24), 15.7 (q, C-25).

Kudinoside A (**10**)

White powder; $[\alpha]^{23}_{D}$: -126.6° (c = 0.05, MeOH); UV λ_{max} (MeOH) nm (log ε): 207 (4.09), 277 (3.85), 255 (3.69); IR ν_{max} (KBr) cm^{-1}: 3398, 2942, 2875, 1732, 1641,

1451, 1377, 1075, 1055; Mp: 195-196°; ESIMS: *m/z* 949.5 $[M+Na]^+$; ^{1}H NMR (C_5D_5N, 400 MHz) δ: 6.34 (1H, br s, Rha H-1''), 5.92 (1H, br s, H-12), 5.11 (1H, d, *J* = 8.0 Hz, Glc H-1'''), 4.87 (1H, d, *J* = 5.6 Hz, Ara H-1'), 3.31 (1H, dd, *J* = 11.6, 4.0 Hz, H-3), 1.66 (3H, s, H-29), 1.64 (3H, d, *J* = 4.4 Hz, Rha H-6''), 1.63 (3H, s, H-27), 1.51 (3H, s, H-30), 1.23 (3H, s, H-23), 1.14 (3H, s, H-24), 0.91 (3H, s, H-26), 0.88 (3H, s, H-25); ^{13}C NMR (C_5D_5N, 100 MHz) δ: 175.5 (s, C-28), 146.4 (s, C-13), 137.5 (s, C-18), 104.8 (d, Ara C-1'), 104.7 (d, Glc C-1'''), 101.9 (d, Rha C-1''), 88.3 (d, C-3), 85.6 (s, C-20), 82.2 (d, Ara C-3'), 78.6 (d, Glc C-5'''), 78.2 (d, Glc C-3'''), 75.0 (d, Glc C-2'''), 74.7 (d, Ara C-2'), 74.3 (s, C-19), 73.9 (d, Rha C-4''), 72.5 (d, Rha C-3''), 72.4 (d, Rha C-2''), 71.4 (d, Glc C-4'''), 70.0 (d, Rha C-5''), 68.2 (d, Ara C-4'), 66.0 (d, C-12), 64.9 (t, Ara C-5'), 62.5 (t, Glc C-6'''), 56.2 (d, C-5), 44.9 (d, C-9), 44.1 (s, C-17), 43.9 (s, C-14), 41.7 (s, C-8), 39.6 (s, C-4), 39.2 (t, C-1), 37.0 (s, C-10), 35.5 (t, C-7), 32.8 (t, C-22), 28.8 (t, C-2), 28.8 (t, C-15), 28.4 (t, C-21), 28.0 (q, C-23), 26.7 (t, C-16), 26.2 (t, C-11), 25.2 (q, C-29), 23.5 (q, C-27), 19.5 (q, C-30), 18.6 (q, Rha C-6''), 18.5 (t, C-6), 18.2 (q, C-26), 16.9 (q, C-24), 16.7 (q, C-25).

Kudinoside D (**11**)

White powder; $[\alpha]^{23}_{D}$: -79.0° (*c* = 0.05, MeOH); UV λ_{max} (MeOH) nm (log ε): 220 (3.77), 259 (4.26); IR ν_{max} (KBr) cm^{-1}: 3431, 2940, 1739, 1646, 1454, 1395, 1073; Mp:

201-202°; ESIMS: *m/z* 907.6 [M-H]$^+$; ^{1}H NMR (C_5D_5N, 400 MHz) δ: 7.52 (1H, dd, *J* = 10.4, 2.0 Hz, H-11), 6.19 (1H, s, Rha H-1''), 5.78 (1H, d, *J* = 10.4, H-12), 5.13 (1H, d, *J* = 7.6, Glc H-1'''), 4.89 (1H, d, *J* = 5.6, Ara H-1'), 3.31 (1H, dd, *J* = 11.6, 4.0, H-3), 1.70 (3H, s, H-29), 1.66 (3H, d, *J* = 6.0 , Rha H-1''), 1.54 (3H, s, H-30), 1.22 (3H, s, H-23), 1.11 (3H, s, H-24), 1.06 (3H, s, H-27), 0.89 (3H, s, H-26), 0.84 (3H, s, H-25); ^{13}C NMR (C_5D_5N, 100 MHz) δ: 175.2 (s, C-28), 140.7 (s, C-13), 135.5 (s, C-18), 128.4 (d, C-12), 127.2 (d, C-11), 104.9 (d, Ara C-1'), 104.8 (d, Glc C-1'''), 102.0 (d, Rha C-1''), 88.2 (d, C-3), 85.9 (s, C-20), 82.3 (d, Ara C-3'), 78.6 (d, Glc C-5'''), 78.3 (d, Glc C-3'''), 75.0 (d, Glc C-2'''), 74.8 (d, Ara C-2'), 74.1 (s, C-19), 73.9 (d, Rha C-4''), 72.5 (d, Rha C-2''), 72.5 (d, Rha C-3''), 71.5 (d, Glc C-4'''), 70.1 (d, Rha C-5''), 68.3 (d, Ara C-4'), 64.9 (t, Ara C-5'), 62.5 (t, Glc C-6'''), 55.3 (d, C-5), 54.5 (d, C-9), 43.8 (s, C-17), 42.2(s, C-8), 42.2(s, C-14), 39.7 (s, C-4), 38.4 (t, C-1), 36.6 (s, C-10), 32.9 (t, C-7), 32.9 (t, C-22), 28.6 (t, C-21), 27.7 (q, C-23), 26.5 (t, C-2), 26.3 (t, C-16), 25.9 (t, C-15), 23.7 (q, C-29), 19.5 (q, C-30), 18.7 (q, C-27), 18.6 (q, Rha C-6''), 18.5 (q, C-25), 18.4 (t, C-6), 16.5 (q, C-26), 16.4 (q, C-24).

Kudinoside C (**12**)

White powder; $[\alpha]^{23}_D$: -86.5° (*c* = 0.05, MeOH); UV λ_{max} (MeOH) nm (log ε): 207 (3.88), 225 (3.67); IR ν_{max} (KBr) cm^{-1}: 3402, 2943, 2874, 1730, 1642, 1452, 1379,

1080; Mp: 193-194°; ESIMS: *m/z* 1087.7 [M-H]$^+$; ^{1}H NMR (C_5D_5N, 400 MHz) δ: 6.40 (1H, br s, Rha H-1''), 5.92 (1H, br s, H-12), 5.27 (1H, d, J = 7.6 Hz, Glc H-1''''), 5.16 (1H, d, J = 7.6 Hz, Glc H-1'''), 4.76 (1H, m, Ara H-1'), 3.29 (1H, dd, J = 11.6, 4.0 Hz, H-3), 1.72 (3H, d, J = 6.0 Hz, Rha H-6''), 1.66 (3H, s, H-29), 1.62 (3H, s, H-27), 1.51 (3H, s, H-30), 1.25 (3H, s, H-23), 1.18 (3H, s, H-24), 0.90 (3H, s, H-26), 0.87 (3H, s, H-25); ^{13}C NMR (C_5D_5N, 100 MHz) δ: 175.4 (s, C-28), 146.3 (s, C-13), 137.4 (s, C-18), 106.4 (d, Glc C-1''''), 105.1 (d, Ara C-1'), 103.1 (d, Glc C-1'''), 100.9 (d, Rha C-1''), 88.4 (d, C-3), 85.7 (s, C-20), 84.8 (d, Glc C-2''''), 83.0 (d, Ara C-3'), 78.8 (d, Glc-5''''), 78.4 (d, Glc C-5'''), 78.3 (d, Glc C-3'''), 78.2 (d, Glc-3''''), 76.1 (d, Glc C-2''''), 74.3 (d, Ara C-2'), 74.2 (s, C-19), 73.9 (d, Rha C-4''), 72.5 (d, Rha C-3''), 72.4 (d, Rha C-2''), 70.8 (d, Glc C-4'''), 70.4 (d, Glc C-4''''), 69.8 (d, Rha C-5''), 69.4 (d, Ara C-4'), 66.0 (d, C-12), 65.8 (t, Ara C-5'), 62.3 (t, Glc C-6'''), 61.8 (t, Glc-6''''), 56.3 (d, C-5), 44.8 (d, C-9), 44.1 (s, C-17), 43.9 (s, C-14), 41.7 (s, C-8), 39.5 (s, C-4), 39.2 (t, C-1), 37.0 (s, C-10), 35.5 (t, C-7), 32.8 (t, C-22), 28.8 (t, C-2), 28.8 (t, C-15), 28.3 (t, C-21), 28.0 (q, C-23), 26.7 (t, C-16), 26.2 (t, C-11), 25.2 (q, C-29), 23.4 (q, C-27), 19.5 (q, C-30), 18.5 (t, C-6), 18.3 (q, Rha C-6''), 18.2 (q, C-26), 17.1 (q, C-24), 16.8 (q, C-25).

Kudinoside F (**13**)

White powder; $[\alpha]^{23}_{D}$: -104.4° (c = 0.05, MeOH); UV λ_{max} (MeOH) nm (log ε): 214

(3.89), 227 (3.81); IR ν_{max} (KBr) cm^{-1}: 3411, 2939, 1729, 1635, 1456, 1366, 1076, 1024; Mp: 261-262°; ESIMS: *m/z* 925.6 $[M-H]^+$; ^1H NMR (C_5D_5N, 400 MHz) δ: 6.22 (1H, br s, Rha H-1''), 5.13 (1H, d, *J* = 7.6 Hz, Glc H-1'''), 4.95 (1H, d, *J* = 7.6 Hz, H-11), 4.86 (1H, d, *J* = 5.6 Hz, Ara H-1'), 3.28 (1H, dd, *J* = 11.6, 4.0 Hz, H-3), 1.81 (3H, s, H-29), 1.65 (3H, d, *J* = 6.0 Hz, Rha H-6''), 1.55 (3H, s, H-30), 1.27 (3H, s, H-27), 1.21 (3H, s, H-23), 1.10 (3H, s, H-24), 0.89 (3H, s, H-26), 0.82 (3H, s, H-25); ^{13}C NMR (C_5D_5N, 100 MHz) δ: 175.4 (s, C-28), 143.5 (s, C-13), 135.6 (s, C-18), 104.9 (d, Ara C-1'), 104.8 (d, Glc C-1'''), 101.9 (d, Rha C-1''), 88.0 (d, C-3), 85.3 (s, C-20), 82.4 (d, Ara C-3'), 78.6 (d, Glc C-5'''), 78.2 (d, Glc C-3'''), 74.9 (d, Glc C-2'''), 74.6 (d, Ara C-2'), 73.9 (d, Rha C-4''), 73.2 (s, C-19), 72.5 (d, Rha C-2''), 72.5 (d, Rha C-3''), 71.5 (d, Glc C-4'''), 71.4 (d, C-11), 70.0 (d, Rha C-5''), 68.3 (d, Ara C-4'), 65.0 (t, Ara C-5'), 62.5 (t, Glc C-6'''), 55.9 (d, C-5), 50.1 (d, C-9), 46.2 (s, C-17), 45.5 (s, C-14), 42.7 (s, C-8), 39.5 (s, C-4), 39.0 (t, C-1), 37.1 (s, C-10), 35.1 (t, C-7), 33.5 (t, C-12), 32.4 (t, C-22), 29.2 (t, C-15), 29.2 (t, C-21), 27.9 (q, C-23), 26.9 (t, C-2), 26.6 (t, C-16), 26.5 (q, C-29), 21.5 (q, C-27), 20.2 (q, C-30), 18.6 (q, Rha C-6''), 18.4 (t, C-6), 17.1 (q, C-26), 16.9 (q, C-25), 16.8 (q, C-24).

Latifolioside H (**14**)

White powder; $[\alpha]^{23}_D$: -80.0° (*c* = 0.05, MeOH); UV λ_{max} (MeOH) nm (log ε): 207

(3.54); IR ν_{max} (KBr) cm^{-1}: 3414, 2937, 1739, 1635, 1454, 1386, 1073, 1030; Mp: 236-237°; ESIMS: *m/z* 1219.8 $[M-H]^+$; ^1H NMR (C_5D_5N, 400 MHz) δ: 6.72 (1H, br s, Rha H-1'''''), 6.27 (1H, d, *J* = 8.0 Hz, Glc H-1''''), 6.23 (1H, br s, Rha H-1''), 5.55 (1H, m, H-12), 5.12 (1H, d, *J* = 8.0 Hz, Glc H-1'''), 4.87 (1H, m, Ara H-1'), 3.27 (1H, d, *J* = 11.2 Hz, H-3), 1.80 (3H, d, *J* = 6.0 Hz, Rha H-6''), 1.65 (3H, m, Rha H-6'''''), 1.65 (3H, s, H-27), 1.16 (3H, s, H-23), 1.12 (3H, s, H-29), 1.10 (3H, s, H-24), 1.10 (3H, s, H-26), 0.87 (3H, s, H-25), 0.85 (3H, s, H-30); ^{13}C NMR (C_5D_5N, 100 MHz) δ: 177.1 (s, C-28), 144.4 (s, C-13), 123.7 (d, C-12), 104.8 (d, Ara C-1'), 104.6 (d, Glc C-1'''), 101.9 (d, Rha C-1''), 101.4 (d, Rha C-1'''''), 95.0 (d, Glc C-1''''), 88.2 (d, C-3), 82.3 (d, Ara C-3'), 81.1 (d, C-19), 79.8 (d, Glc C-3''''), 78.9 (d, Glc C-5''''), 78.5 (d, Glc-5'''), 78.2 (d, Glc C-3'''), 75.3 (d, Glc C-2''''), 74.9 (d, Glc C-2'''), 74.7 (d, Ara C-2'), 73.9 (d, Rha C-4''), 73.9 (d, Rha C-4'''''), 72.5 (d, Rha C-3''), 72.4 (d, Rha C-4''), 72.4 (d, Rha C-3'''''), 72.2 (d, Rha C-2'''''), 71.3 (d, Glc C-4''''), 71.3 (d, Glc-4'''), 70.0 (d, Rha C-5''), 69.7 (d, Rha C-5'''''), 68.3 (d, Ara C-4'), 64.9 (t, Ara C-5'), 62.4 (t, Glc C-6'''), 61.9 (d, Glc C-6''''), 56.1 (d, C-5), 48.2 (d, C-9), 46.6 (s, C-17), 44.9 (d, C-18), 42.3 (s, C-14), 40.1 (s, C-8), 39.5 (s, C-4), 39.0 (t, C-1), 37.1 (s, C-10), 35.4 (s, C-20), 33.3 (t, C-22), 32.6 (t, C-7), 29.5 (t, C-15), 28.9 (t, C-21), 28.7 (q, C-29), 28.0 (q, C-23), 27.8 (t, C-16), 26.6 (t, C-2), 24.6 (q, C-30), 24.5 (q, C-27), 24.1 (t, C-11), 18.6 (t, C-6), 18.6 (q, Rha C-6'''''), 18.5 (q, Rha C-6''), 17.5 (q, C-26), 16.9 (q, C-24), 15.6 (q, C-25).

Latifolioside G (**15**)

White powder; $[\alpha]^{23}_{D}$: -113.7° (c = 0.05, MeOH); UV λ_{max} (MeOH) nm (log ε): 206 (3.49); IR ν_{max} (KBr) cm^{-1}: 3414, 2932, 1738, 1638, 1456, 1384, 1075, 1028; Mp: 203-204°; ESIMS: *m/z* 1219.8 $[M-H]^{+}$; ^{1}H NMR (C_5D_5N, 400 MHz) δ: 6.74 (1H, br s, Rha H-1'''''), 6.19 (1H, br s, Rha H-1''), 6.18 (1H, d, J = 8.4 Hz, Glc H-1''''), 5.58 (1H, m, H-12), 5.11 (1H, d, J = 7.6 Hz, Glc H-1'''), 4.84 (1H, m, Ara H-1'), 3.25 (1H, dd, J = 11.2, 2.8 Hz, H-3), 1.78 (3H, d, J = 6.0 Hz, Rha H-6''), 1.72 (3H, s, H-27), 1.63 (3H, d, J = 6.0 Hz, Rha H-6'''''), 1.42 (3H, s, H-29), 1.13 (3H, s, H-23), 1.13 (3H, s, H-26), 1.08 (3H, s, H-25), 1.08 (3H, s, H-30), 0.88 (3H, s, H-24); ^{13}C NMR (C_5D_5N, 100 MHz) δ: 176.8 (s, C-28), 139.2 (s, C-13), 128.2 (d, C-12), 104.7 (d, Ara C-1'), 104.5 (d, Glc C-1'''), 101.8 (d, Rha C-1''), 101.3 (d, Rha C-1'''''), 94.8 (d, Glc C-1''''), 88.2 (d, C-3), 82.2 (d, Ara C-3'), 80.0 (d, Glc C-3''''), 78.9 (d, Glc C-5''''), 78.4 (d, Glc-5'''), 78.1 (d, Glc C-3'''), 75.1 (d, Glc C-2''''), 74.8 (d, Glc C-2'''), 74.6 (d, Ara C-2'), 73.8 (d, Rha C-4''), 73.7 (d, Rha C-4'''''), 72.6 (d, Rha C-3''), 72.5 (d, Rha C-4''), 72.4 (d, Rha C-3'''''), 72.2 (d, Rha C-2'''''), 71.4 (d, Glc C-4''''), 71.3 (d, Glc-4'''), 69.9 (d, Rha C-5''), 69.6 (d, Rha C-5'''''), 68.2 (d, Ara C-4'), 64.8 (t, Ara C-5'), 62.4 (t, Glc C-6'''), 62.2 (d, Glc C-6''''), 56.1 (d, C-5), 54.6 (d, C-18), 48.6 (s, C-17), 47.6 (d, C-9), 42.1 (s, C-14), 41.8 (d, C-20), 40.4 (s, C-8), 39.5 (s, C-4), 39.1 (t, C-1), 37.4 (t, C-22), 36.9 (s, C-10),

33.6 (t, C-7), 29.6 (t, C-15), 28.0 (q, C-23), 27.0 (q, C-29), 26.6 (t, C-16), 26.6 (t, C-21), 26.0 (t, C-2), 24.3 (q, C-27), 24.0 (t, C-11), 18.6 (t, C-6), 18.6 (q, Rha C-6'''''), 18.5 (q, Rha C-6''), 17.3 (q, C-26), 16.9 (q, C-30), 16.6 (q, C-24), 15.7 (q, C-25).

β-sitosterol (**16**)

White powder; $[\alpha]^{23}_{D}$: -86.5° (c = 0.05, $CHCl_3$); UV λ_{max} (MeOH) nm (log ε): 245 (2.84); IR ν_{max} (KBr) cm^{-1}: 3427, 2936, 2868, 1463, 1380, 1051, 1023; Mp: 141-142°; R_f = 0.55 ($CHCl_3$: MeOH = 40 : 1, v/v); ^{1}H NMR (C_5D_5N, 400 MHz) δ: 5.35 (1H, m, H-6), 3.53 (1H, m, H-3), 1.01 (3H, s, H-29), 0.92 (3H, d, J = 6.4 Hz, H-19), 0.85 (3H, t, J = 7.2 Hz, H-24), 0.84 (3H, d, J = 7.2 Hz, H-26), 0.81 (3H, d, J = 7.2 Hz, H-27), 0.69 (3H, s, H-28). ^{13}C NMR (C_5D_5N, 100 MHz) δ: 140.8 (s, C-5), 121.7 (d, C-6), 71.8 (d, C-3), 56.8 (d, C-14), 56.0 (d, C-17), 50.1 (d, C-9), 45.8 (d, C-22), 42.3 (t, C-4), 42.3 (d, C-13), 39.8 (t, C-12), 37.2 (t, C-1), 36.5 (s, C-10), 36.1 (d, C-18), 33.9 (t, C-20), 31.9 (t, C-7), 31.9 (d, C-8), 31.7 (t, C-2), 29.1 (d, C-25), 28.0 (t, C-16), 26.1 (t, C-21), 24.3 (t, C-15), 23.1 (t, C-23), 21.1 (t, C-11), 19.8 (q, C-19), 19.4 (q, C-26), 19.0 (q, C-27), 18.8 (q, C-28), 12.0 (q, C-24), 11.9 (q, C-29).

Menisdaurin D (**17**)

Colorless plates; $[\alpha]^{23}_{D}$: -141.0° (c = 0.05, MeOH); UV λ_{max} (MeOH) nm (log ε): 214

(4.01); IR ν_{max} (KBr) cm^{-1}: 3340, 2950, 2914, 2218, 1641, 1118, 1080, 1064, 1044; Mp: 165-166°; ESIMS: *m/z* 338.2 $[M+Na]^+$; ^1H NMR (DMSO-d_6, 400 MHz) δ: 5.50 (1H, s, H-7), 4.48 (1H, dd, *J* = 8.4, 4.0 Hz, H-6), 4.31 (1H, d, *J* = 7.2, H-1'), 3.73 (1H, m, H-4), 3.65 (1H, d, *J* = 11.2 Hz, H-6'a), 3.43 (1H, dd, *J* = 11.2, 5.6 Hz, H-6'b), 3.02~3.18 (4H, m, H-2'~H-5'), 2.48 (1H, m, H-2a), 2.11 (2H, m, H-2b, H5a), 1.77 (1H, m, H-3a), 1.60 (1H, m, H-5b), 1.41 (1H, m, H-3b). ^{13}C NMR (DMSO-d_6, 100 MHz) δ: 165.7 (s, C-1), 117.8 (s, C-8), 101.5 (d, C-1'), 93.5 (d, C-7), 78.0 (d, C-5'), 77.8 (d, C-3'), 75.0 (d, C-6), 74.0 (d, C-2'), 71.1 (d, C-4'), 66.3 (d, C-4), 62.2 (t, C-6'), 40.7 (t, C-5), 36.3 (t, C-3), 30.2 (t, C-2).

Menisdaurin (**18**)

Colorless plate; $[\alpha]^{23}_{D}$: -204.2° (*c* = 0.05, MeOH); UV λ_{max} (MeOH) nm (log ε): 206 (3.71), 258 (4.39); IR ν_{max} (KBr) cm^{-1}: 3343, 2914, 2211, 1648, 1627, 1074, 1059, 1020; Mp: 173-174°; ESIMS: *m/z* 336.3 $[M+Na]^+$; ^1H NMR (CD_3OD, 400 MHz) δ: 6.32 (1H, d, *J* = 10.0 Hz, H-2), 6.24 (1H, dd, *J* = 10.0, 3.2 Hz, H-3), 5.58 (1H, s, H-7), 4.96 (1H, ddd, *J* = 8.0, 2.4, 1.2 Hz, H-6), 4.58 (1H, d, *J* = 6.4 Hz, H-1'), 4.40 (1H, m, H-4), 3.92 (1H, dd, *J* = 11.6, 2.0 Hz, H-6'a), 3.70 (1H, dd, *J* = 11.6, 6.0 Hz, H-6'b), 3.29~3.45 (4H, m, H-2'~H-5'), 2.30 (1H, ddd, *J* = 13.2, 5.2, 4.4 Hz, H-5a), 2.06 (1H, ddd, *J* = 13.2, 8.0, 6.2 Hz, H-5b). ^{13}C NMR (CD_3OD, 100 MHz) δ: 157.1 (s, C-1),

140.6 (d, C-3), 127.7 (d, C-2), 118.0 (s, C-8), 101.5 (d, C-1'), 96.8 (d, C-7), 78.1 (d, C-5'), 77.9 (d, C-3'), 74.4 (d, C-2'), 72.4 (d, C-6), 71.7 (d, C-4'), 65.3 (d, C-4), 63.1 (t, C-6'), 36.1 (t, C-5).

α-Kudinlactone (**11-1**)

White powder; $[\alpha]^{23}_{D}$: 87.4° (*c* = 0.05, MeOH); UV λ_{max} (MeOH) nm (log ε): 220 (3.63), 259 (4.11); IR ν_{max} (KBr) cm^{-1}: 3450, 2938, 2868, 1721, 1622, 1454, 1383, 1073, 1047; Mp: 231-232°; ESIMS: *m/z* 491.4 $[M+Na]^{+}$; ^{1}H NMR (C_5D_5N, 400 MHz) δ: 7.55 (1H, dd, *J* = 10.4, 2.8 Hz, H-11), 5.86 (1H, d, *J* = 10.4, H-12), 3.47 (1H, dd, *J* = 10.4, 5.2, H-3), 1.70 (3H, s, H-29), 1.54 (3H, s, H-30), 1.24 (3H, s, H-23), 1.05 (3H, s, H-27), 1.03 (3H, s, H-24), 0.96 (3H, s, H-25), 0.89 (3H, s, H-26); ^{13}C NMR (C_5D_5N, 100 MHz) δ: 175.2 (s, C-28), 140.8 (s, C-13), 135.0 (s, C-18), 128.5 (d, C-12), 127.1 (d, C-11), 85.9 (s, C-20), 80.0 (d, C-3), 74.1 (s, C-19), 55.1 (d, C-5), 54.6 (d, C-9), 43.8 (s, C-17), 42.2 (s, C-8), 42.2 (s, C-14), 39.5 (s, C-4), 38.4 (t, C-1), 37.0 (s, C-10), 33.0 (t, C-7), 32.9 (t, C-22), 28.5 (t, C-21), 28.4 (q, C-23), 27.9 (t, C-2), 26.3 (t, C-16), 25.9 (t, C-15), 23.7 (q, C-29), 19.5 (q, C-30), 18.7 (q, C-27), 18.6 (t, C-6), 18.4 (q, C-25), 16.5 (q, C-26), 15.9 (q, C-24).

Kudinchagenin I (**11-2**)

White powder; [α] ^{23}$_{D}$: 106.4° (c = 0.05, MeOH); UV λ_{max} (MeOH) nm (log ε): 208 (4.45), 293 (4.64); IR ν_{max} (KBr) cm^{-1}: 3434, 2937, 2869, 1746, 1627, 1457, 1078, 1048; Mp: 244-245°; ESIMS: m/z 473.4 $[M+Na]^+$; ^{1}H NMR (C_5D_5N, 400 MHz) δ: 6.94 (1H, dd, J = 10.4, 2.4 Hz, H-11), 5.89 (1H, d, J = 10.4, H-12), 5.44 (1H, s, H-29a),5.38 (1H, s, H-29b), 3.48 (1H, dd, J = 9.2, 6.8, H-3), 1.55 (3H, s, H-30), 1.24 (3H, s, H-23), 1.10 (3H, s, H-27), 1.02 (3H, s, H-24), 0.87 (3H, s, H-25), 0.76 (3H, s, H-26). ^{13}C NMR (C_5D_5N, 100 MHz) δ: 175.0 (s, C-28), 146.2 (s, C19), 141.3 (s, C-13), 131.5 (d, C-12), 129.2 (s, C-18), 125.8 (d, C-11), 111.7 (t, C-29), 83.2 (s, C-20), 78 (d, C-3), 55.1 (d, C-5), 54.1 (d, C-9), 45.3 (s, C-17), 42.8 (s, C-8), 41.9 (s, C-14), 39.5 (s, C-4), 38.4 (t, C-1), 36.9 (s, C-10), 35.5 (t, C-22), 32.9 (t, C-7), 31.4 (t, C-21), 28.4 (q, C-23), 27.9 (t, C-2), 25.6 (t, C-16), 25.4 (t, C-15), 21.7 (q, C-30), 20.2 (q, C-27), 18.5 (t, C-6), 18.3 (q, C-25), 16.7 (q, C-26), 16.0 (q, C-24).

Siaresinolic acid (**14-1**)

White powder; [α] ^{23}$_{D}$: 46.5° (c = 0.05, MeOH); UV λ_{max} (MeOH) nm (log ε): 208 (4.02); IR ν_{max} (KBr) cm^{-1}: 3430, 2938, 2872, 1702, 1629, 1454, 1385, 1073, 1024; Mp: 271-272°; ESIMS: m/z 471.5 $[M-H]^+$; ^{1}H NMR (C_5D_5N, 400 MHz) δ:, 5.60 (1H, m, H-12), 3.66 (2H, m, H-18, H-19), 3.46 (1H, dd, J = 11.2, 5.2 Hz, H-3), 1.68 (3H, s, H-27), 1.26 (3H, s, H-23), 1.21 (3H, s, H-29), 1.14 (3H, s, H-30), 1.10 (3H, s, H-26),

1.05 (3H, s, H-24), 0.94 (3H, s, H-25); ^{13}C NMR (C_5D_5N, 100 MHz) δ: 180.9 (s, C-28), 144.9 (s, C-13), 123.5 (d, C-12), 81.3 (d, C-19), 78.1 (d, C-3), 55.9 (d, C-5), 48.4 (d, C-9), 46.1 (s, C-17), 44.8 (d, C-18), 42.1 (s, C-14), 40.0 (s, C-8), 39.4 (s, C-4), 38.8 (t, C-1), 37.5 (s, C-10), 35.7 (s, C-20), 33.7 (t, C-7), 33.4 (t, C-22), 29.2 (t, C-21), 29.2 (q, C-23), 28.9 (t, C-15), 28.8 (q, C-29), 28.4 (t, C-2), 28.1 (t, C-16), 24.9 (q, C-30), 24.8 (q, C-27), 24.2 (t, C-11), 19.0 (t, C-6), 17.5 (q, C-26), 16.5 (q, C-24), 15.5 (q, C-25).

Randialic acid B (**15-1**)

White powder; $[\alpha]^{23}_{D}$: 59.7° (c = 0.05, MeOH); UV λ_{max} (MeOH) nm (log ε): 224 (4.04); IR ν_{max} (KBr) cm^{-1}: 3440, 2932, 2868, 1696, 1456, 1384, 1276, 1084, 1033; Mp: 263-264°; ESIMS: m/z 453.4 [M-H]$^{+}$; ^{1}H NMR (C_5D_5N, 400 MHz) δ: 5.75 (1H, m, H-12), 3.49 (1H, dd, J = 10.0, 6.0 Hz, H-3), 1.89 (3H, s, H-29), 1.26 (3H, s, H-23), 1.16 (3H, s, H-27), 1.13 (3H, d, J = 6.8 Hz, H-30), 1.07 (3H, s, H-26), 1.05 (3H, s, H-24), 0.91 (3H, s, H-25); ^{13}C NMR (C_5D_5N, 100 MHz) δ: 178.6 (s, C-28), 139.5 (s, C-13), 134.7 (s, C-19), 126.0 (d, C-12), 123.7 (s, C-18), 78.1 (d, C-3), 56.0 (d, C-5), 49.8 (s, C-17), 48.3 (d, C-9), 45.0 (s, C-14), 39.5 (s, C-4), 39.4 (t, C-1), 39.3 (s, C-8), 37.2 (s, C-10), 35.6 (t, C-7), 35.1 (t, C-22), 34.8 (d, C-20), 31.9 (t, C-21), 29.2 (t, C-15), 28.8 (q, C-23), 28.2 (t, C-2), 27.0 (t, C-16), 23.5 (t, C-11), 22.1 (q, C-27), 19.6

(q, C-29), 18.9 (q, C-30), 18.8 (t, C-6), 18.3 (q, C-26), 16.7 (q, C-24), 16.3 (q, C-25).

Sanguisorbigenin (**15-2**)

White powder; $[\alpha]^{23}_{D}$: 70.4° (*c* = 0.05, MeOH); UV λ_{max} (MeOH) nm (log ε): 208 (4.08), 250 (3.33); IR ν_{max} (KBr) cm^{-1}: 3430, 2931, 1698, 1454, 1383, 1255, 1103, 1029; Mp: 251-252°; ESIMS: *m*/*z* 453.4 $[M-H]^{+}$; ^{1}H NMR (C_5D_5N, 400 MHz) δ: 5.72 (1H, m, H-12), 3.67 (1H, br s, H-18), 3.48 (1H, dd, *J* = 10.4, 6.0 Hz, H-3), 1.72 (3H, s, H-29), 1.64 (3H, s, H-30), 1.22 (3H, s, H-23), 1.16 (3H, s, H-27), 1.05 (3H, s, H-26), 1.04 (3H, s, H-25), 0.92 (3H, s, H-24); ^{13}C NMR (C_5D_5N, 100 MHz) δ: 179.8 (s, C-28), 138.5 (s, C-13), 129.0 (s, C-19), 127.3 (d, C-12), 123.9 (s, C-20), 78.1 (d, C-3), 56.0 (d, C-5), 50.7 (d, C-18), 48.2 (d, C-9), 47.0 (s, C-17), 43.8 (s, C-14), 39.6 (s, C-4), 39.4 (s, C-8), 39.3 (t, C-1), 37.3 (s, C-10), 34.3 (t, C-7), 33.5 (t, C-22), 28.8 (q, C-23), 28.7 (t, C-21), 28.7 (t, C-15), 28.2 (t, C-2), 24.0 (t, C-16), 23.7 (t, C-11), 22.2 (q, C-27), 20.5 (q, C-30), 18.7 (t, C-6), 18.0 (q, C-26), 17.4 (q, C-29), 16.7 (q, C-24), 16.1 (q, C-25).

Supplementary information S2

The enzyme inhibition assay modified according to the methods described before.[16-17] In general, 25 μl of enzyme solution was added to 50 μl of drug solution in 96-well plate. After gentle shake for 5 min in the dark, 25 μl of substrate solution was add to the mixture and incubated until the chlorogenic or fluorogenic absorbance was measured by automatic microplate readers (Thermo Labsystems Multiskan Ascent and HIDEX Multilabel Reader). The blank solution consists of 50 μL of buffer and 50 μL of drug solution. Elaspol, CG inhibitor (CALBIOCHEM, 219372) and AEBSF (Enzo, ALX-270-022) were used as positive controls.[19-22] Inhibition of enzyme activity (%) was determined by the following equation:

$$\frac{\text{Abs. diff. between control and blank} - \text{Abs. diff. between reaction solution and blank}}{\text{Abs. diff. between control and blank}} \times 100\%$$

The condition of enzymatic reactions such as substrates, buffers, detection wave and final working concentration were further described below.

Human neutrophil elastase

Human neutrophil elastase (Enzo, BML-SE284) and substrate (Enzo, BML-P213) were prepared in buffer A. Tested agents were prepared in buffer B. The final

concentrations of enzyme, substrate are 50 nM and 125 μM, respectively. The absorption at 405 nm was measured 15 min after the enzymatic reaction began.

Human cathesin G

Human cathepsin G (Enzo, BML-SE283), substrate (Enzo, BML-P141) and tested agents were all prepared in prepared in buffer C. The final concentrations of enzyme, substrate are 100 nM and 200 μM, respectively. The absorption at 405 nm was measured after the enzymatic reaction began at intervals of 30 min for 180 min.

Human proteinase 3

Human proteinase 3 (Merk, 539483), substrate (Enzo, BML-P303) and tested agents were all prepared in prepared in buffer D. The final concentrations of enzyme, substrate are 10 nM and 100 μM, respectively. The absorption at 405 nm was measured 60 min after the enzymatic reaction began.

Human thrombin

Human thrombin (Sigma-aldrich, T7009-100KU) and substrate (Merck, 605211) were prepared in buffer A. Tested agents were prepared in buffer B. The final concentrations of enzyme, substrate are 10 nM and 25 μM, respectively. The

excitation /emission waves at 340/460 nm was measured 30 min after the enzymatic reaction began.

Bovine chymotrypsin

Bovine chymotrypsin (Sigma-Aldrich, C4129) and substrate (Sigma-Aldrich, S9761) were prepared in buffer A. Tested agents were prepared in buffer B. The final concentrations of enzyme, substrate are 5 nM and 5 μM, respectively. The ex/em waves at 340/460 nm was measured 30 min after the enzymatic reaction began.

Buffers

Buffer A: 20mM Tris-HCL, pH 7.4, 0.1% NaN_3

Buffer B: 20mM Tris-HCL, pH 7.4, 0.1% NaN_3, 5 mM $CaCl_2$

Buffer C: 100 mM Tris-HCl, 1.6 M NaCl, pH 7.5

Buffer D: 100 mM HEPES, pH 7.5, 500 mM NaCl, 10% DMSO, 170 μM

Reference

1. Shen, D. *et al*. LC-MS method for the simultaneous quantitation of the anti-inflammatory constituents in oregano (*Origanum* species). *J. Agric. Food Chem*. **58**, 7119-7125 (2010).
2. Chaturvedula, V. S. P & Prakash, I. Isolation of stigmasterol and β-sitosterol from the dichloromethane extract of *Rubus suavissimus*. *Int. Curr. Pharmaceut. J.* **1**, 239-242 (2012).
3. Yogo, M., Ishiguro, S., Murata, H. & Furukawa, H. Coclauril, a nonglucosidic 2-cyclohexen-1-ylideneacetonitrile, form *Cocculus lauriforium* DC. *Chem. Pharm. Bull*. **38**, 225-226 (1990).
4. Ueda, K., Yasutomi, K. & Mori, I. Structure of a new cyanoglucoside from *Ilex warburgii* Loesn. *Chem. Lett*. **12**, 149-150 (1983).
5. Seigler, D. S. *et al*. Cyanogenic glycosides and menisdaurin from *Guazuma ulmifolia*, *Ostrya virginiana*, *Tiquilia plicata*, and *Tiquilia canescens*. *Phytochemistry* **66**, 1567-1580 (2005).
6. Yi, X. X. *et al*. Four new chclohexylideneacetonitrile derivatives from the hypocotyls of mangrove (*Bruguiera gymnorrhiza*). *Molecules* **20**, 14565-14575 (2015).
7. Basnet, P., Matsushige, K., Hase, K., Kadota, S. & Namba, T. Four di-*O*-caffeoyl

quinic acid derivatives from propolis. Potent Hepatoprotective activity in experimental liver injury models. *Biol. Pharm. Bull.* **19**, 1479-1484 (1996).

8. Chen, J. *et al*. Caffeoylquinic acid derevatives isolated from the aerial parts of *Gynura divaricata* and their yeast α-glucosidase and PTP1B inhibitory activity. *Fitoterapia* **99**, 1-6 (2014).
9. Ouyang, M. A., Liu, Y. Q., Wang, H. Q. & Yang, C. R. Triterpenoid saponins from *Ilex latifolia*. *Phytochemistry* **49**, 2483-2486 (1998).
10. Ouyang, M. A., Yang, C. R., Chen, Z. Y. & Wang, H. Q. Triterpenes and triterpenoid glycosides from the leaves of *Ilex kudincha*. *Phytochemistry* **41**, 871-877 (1996).
11. Ouyang, M. A., Wang, H. Q., Chen, Z. L. & Yang, C. R. Triterpenoid glycosides from *Ilex kudincha*. *Phytochemistry* **43**, 443-445 (1996).
12. Mimaki, Y. *et al*. Triterpene glycosides from the roots of *Sanguisorba officinalis*. *Phytochemistry* **57**, 773-779 (2001).
13. Wen, Y. X., Liang, X. Y. & Cheng, G. R. Structural identification of kudinchagenin I. *Acta Bot. Sin.* **41**, 206-208 (1999).
14. Satake, T., Kamiya, K., An, Y., Oishi, T. & Yamamoto, J. The anti-thrombotic active constituents from *Centella asiatica*. *Biol. Pharm. Bull.* **30**, 935-940 (2007).

15. Zhao, Y. *et al*. UFLC/MS-IT-TOF guided isolation of anti-HBV active chlorogenic acid analogues from *Artemisia capillaris* as a traditional Chinese herb for the treatment of hepatitis. *J*. *Ethnopharmacol*. **156**, 147-154 (2014).

16. Stevens, T. *et al*. AZD9668: pharmacological characterization of a novel oral inhibitor of neutrophil elastase. *J*. *Pharmacol*. *Exp*. *Ther*. **339**, 313-320 (2011).

17. Kam, C. M. *et al*. Substrate and inhibitor studies on proteinase 3. *FEBS Lett*. **297**, 119-123 (1992).

18. Wang, M. *et al*. Therapeutic effects of pyroolidine dithiocarbamate on acute lung injury in rabbits. *J*. *Transl*. *Med*. **9**, 61 (2011). DOI: 10.1186/1479-5876-9-61.

19. Kawabata, K. *et al*. ONO-5046, a novel inhibitor of human neutrophil elastase. *Biochem*. *Biophys*. *Res*. *Commun*. **177**, 814-820 (1991).

20. Greco, M. N. *et al*. Nonpeptide inhibitors of cathepsin G: optimization of a novel β-ketophosphonic acid lead by structure-based drug design. *J*. *Am*. *Chem*. *Soc*. **124**, 3810-3811 (2002).

21. Powers, J. C. *et al*. Irreversible inhibitors of serine, systeine, and threonine proteases. *Chem*. *Rev*. **102**, 4639-4750 (2002).

22. Rafael, B. R., Jorge, O. & Jorge, E. G. M. Evaluation of two inhibitors of invasion: LY311727 [3-(3-acetamide-1-benzyl-2-ethyl-indolyl-5-oxy)propane phsphonic acid] and AEBSF [4-(2-aminoethyl)-benzenesulphonyl fluoride] in

acute murine toxoplasmosis. *J. Antimicrob. Chemother.* **49**, 871-874 (2002).
